# Population Pharmacokinetics and Pharmacodynamics of Paracetamol in Malaysian Patients With *Plasmodium knowlesi* Malaria

**DOI:** 10.1002/psp4.70283

**Published:** 2026-06-22

**Authors:** Thanaporn Wattanakul, Daniel J. Cooper, Katherine Plewes, Matthew J. Grigg, Giri S. Rajahram, Timothy William, Arjen M. Dondorp, Michael D. Edstein, Geoffrey W. Birrell, Nicholas M. Anstey, Richard M. Hoglund, Bridget E. Barber, Joel Tarning

**Affiliations:** ^1^ Mahidol Oxford Tropical Medicine Research Unit, Faculty of Tropical Medicine Mahidol University Bangkok Thailand; ^2^ Centre for Tropical Medicine and Global Health, Nuffield Department of Medicine Oxford University Oxford UK; ^3^ Menzies School of Health Research and Charles Darwin University Darwin Northern Territory Australia; ^4^ Infectious Diseases Society Sabah‐Menzies School of Health Research Clinical Research Unit Kota Kinabalu Sabah Malaysia; ^5^ Department of Medicine University of Cambridge School of Clinical Medicine Cambridge UK; ^6^ University of British Columbia Vancouver British Columbia Canada; ^7^ Institute for Health Research University of Notre Dame Australia Fremantle Western Australia Australia; ^8^ Department of Medicine Queen Elizabeth Hospital II, Ministry of Health Malaysia Kota Kinabalu Malaysia; ^9^ Subang Jaya Medical Centre Subang Jaya Selangor Malaysia; ^10^ Department of Drug Evaluation Australian Defence Force Malaria and Infectious Disease Institute Brisbane Queensland Australia; ^11^ QIMR Berghofer Brisbane Australia

**Keywords:** paracetamol, pharmacodynamics, pharmacokinetics, *Plasmodium knowlesi* malaria, renoprotection

## Abstract

Paracetamol may improve renal function in patients with severe *Plasmodium knowlesi* malaria, particularly in those with acute kidney injury and hemolysis, via inhibition of cell‐free hemoglobin mediated oxidative kidney damage. We developed a population pharmacokinetic/pharmacodynamic (PK/PD) model to assess effects of paracetamol on creatinine, hepatotoxicity, fever clearance, and parasite clearance among Malaysian patients with predominantly non‐severe knowlesi malaria using data from the PACKNOW trial (Clinical Trials Registration: NCT03056391). A total of 372 patients were included in the PK/PD analyses (paracetamol: *n* = 183, control: *n* = 189). Paracetamol PK was described using a prior PK model published in patients with falciparum malaria. The PK/PD demonstrated that higher paracetamol exposures were associated with a faster decline in both creatinine and fever clearance time, supporting its renoprotective and antipyretic effects. Increased paracetamol exposure was not associated with hepatotoxicity or serious adverse events, despite a weak positive association with liver transaminases over time. No significant relationship was observed between paracetamol exposure and parasite clearance. Overall, these findings highlight an exposure‐response relationship for paracetamol and a decline in creatinine, supporting its use as a renoprotective drug in treating *Plasmodium knowlesi* malaria.

## Study Highlights

**What is the current knowledge on the topic?:**

- Paracetamol has the potential for renoprotective effects in malaria, possibly by reducing oxidative kidney injury mediated by cell‐free hemoglobin, but detailed exposure‐response data in *Plasmodium knowlesi* malaria are limited.

**What question did this study address?:**

- Is the pharmacokinetic exposure of paracetamol associated with improvements in renal function, fever clearance, liver safety, and parasite clearance in Malaysian patients with *Plasmodium knowlesi* malaria?

**What does this study add to our knowledge?:**

- Using population PK/PD modeling in patients with *Plasmodium knowlesi* malaria (*n* = 372), higher paracetamol exposure was associated with faster creatinine decline and fever clearance, without clinically significant hepatotoxicity or effects on parasite clearance.

**How might this change drug discovery, development, and/or therapeutics?:**

- These findings support the renoprotective and antipyretic effects of paracetamol in malaria and support a wider uptake of its use for these purposes in severe malaria.

## 1 Introduction

Acute kidney injury (AKI) among patients with severe falciparum and knowlesi malaria is a frequent complication and is associated with mortality [1, 2, 3, 4, 5]. Among children with severe falciparum malaria, AKI has been associated with long‐term neurocognitive deficits [6, 7], behavioral problems [8], and chronic kidney disease [6]. Current management strategies have been limited primarily to restrictive fluid therapy, nephrotoxin avoidance and treatment of progressive AKI with renal replacement therapy (RRT); therefore, preventing AKI is essential to reducing morbidity and mortality. Use of paracetamol for this purpose has been limited.

The pathophysiology of malaria‐associated AKI involves oxidative tissue damage mediated by plasma cell‐free hemoglobin (CFH) released during the inherent intravascular hemolysis related to *Plasmodium* species infection [9, 10, 11]. After CFH intraerythrocytic release, ferrous and ferric hemoglobin deplete haptoglobin and hemopexin, resulting in ferric heme oxidization to ferryl heme and globin radicals that trigger lipid peroxidation and kidney damage [10, 12, 13, 14, 15]. Paracetamol has been demonstrated to disrupt this cascade by reducing ferryl heme levels and inhibiting the formation of harmful globin radicals [16]. Paracetamol was shown to mitigate myoglobin‐related renal toxicity in a pre‐clinical model [16], and to lower the risk of AKI requiring RRT in a retrospective study of adults with rhabdomyolysis [17]. A randomized controlled trial (RCT) of paracetamol in adults with sepsis and elevated CFH showed a reduction in oxidative stress and improved renal function [18]. Furthermore, an RCT of paracetamol in Bangladeshi adults with moderate and severe falciparum malaria found that paracetamol improved kidney function and reduced the risk of developing AKI; this renoprotective effect was most pronounced in patients with elevated plasma CFH [19]. The pharmacokinetic/pharmacodynamic (PK/PD) analysis of paracetamol in the Bangladesh study showed that a higher paracetamol exposure increased the probability of an improvement in creatinine over the first 72 h.

In a large RCT of Malaysian patients with predominantly uncomplicated *Plasmodium knowlesi* malaria, regular paracetamol dosing was not associated with renoprotection among the entire study cohort [20]. However, among the pre‐specified subgroups of severe malaria, and those with AKI and hemolysis, regularly dosed paracetamol was associated with a greater proportional reduction in creatinine at 72 h. The current analysis describes the PK of paracetamol in Malaysian patients with *Plasmodium knowlesi* and assesses the PD effects of paracetamol on creatinine, hepatotoxicity, fever clearance, and parasite clearance.

## 2 Methods

### 2.1 Study Design and Patients

This PK/PD analysis was pre‐specified within the PACKNOW trial [20, 21] (Effect of Regularly Dosed Paracetamol vs. No Paracetamol on Renal Function in *Plasmodium knowlesi* Malaria), a two‐arm, open‐label, randomized controlled study undertaken at one tertiary and three district hospitals in Sabah, Malaysia. Hospitalized patients aged ≥ 5 years with microscopy‐confirmed *Plasmodium knowlesi* malaria, who had recent fever, were within 18 h of initiating antimalarial therapy, and provided written informed consent, were enrolled. Patients who were pregnant, had a contraindication or allergy to paracetamol, known cirrhosis, consumed more than six standard alcoholic drinks per day, or lacked PCR‐confirmed *Plasmodium knowlesi* monoinfection were excluded. Patients fulfilling WHO research criteria for severe knowlesi malaria were classified as severe malaria [22], and the remainder as non‐severe malaria. Patients were randomized (1:1 ratio) to receive either paracetamol 1 g orally every 6 h for 72 h or no paracetamol, together with standard antimalarial treatment. AKI was classified using the Kidney Disease: Improving Global Outcomes (KDIGO) criteria [23], excluding urine output criteria. Serial clinical, biochemical, parasitological, and safety assessments were performed. Plasma paracetamol concentrations were measured using intensive or sparse sampling strategies. The intensive schedule included pre‐dose and post‐dose samples at 0.5, 1.5, 2.5, and 4 h after the first dose, with additional samples every 6 h for 72 h and at 72.5, 73.5, 74.5, 76, 78, and 84 h. The sparse schedule consisted of samples collected at enrollment and every 6 h for 72 h. The samples were quantified using validated LC–MS/MS methods [24], full details of the study procedures are provided in the Supplementary Files S1. The study was approved by the Malaysian Research Ethics Committee (NMRR‐16‐356‐29088) and the Ethics Committee of Menzies School of Health Research (2016‐2544), with recognition from the Australian Departments of Defense and Veterans' Affairs Human Research Ethics Committee (142‐19). The trial was registered at ClinicalTrials.gov (NCT03056391).

### 2.2 Population Pharmacokinetic Modeling of Paracetamol

The population PK properties of paracetamol was evaluated using a nonlinear mixed‐effects modeling approach (NONMEM, version 7.4, Icon Development Solution, Ellicott City, MD). Most plasma samples (81.4%) were collected as trough concentrations. This resulted in substantial variability in observed paracetamol concentrations in this study, potentially causing model misspecification and model instability using a standard modeling approach. The PRIOR subroutine ($PRIOR NWPRI) in NONMEM was implemented by using information from a previously published model to stabilize the estimation of the PK parameters of paracetamol. Three candidate prior models were identified from the literature based on their structural similarity to the expected pharmacokinetic behavior of paracetamol in the target population [25, 26, 27]. The suitability of each candidate model was evaluated by applying each prior model to the current dataset with all parameters fixed to their reported estimates without re‐estimation (MAXEVAL = 0) using the following assessments: objective function value (OFV) comparison, visualization of the η‐distributions for parameters with inter‐individual variability, and external visual predictive checks (VPCs). The selected prior model was a PK study of paracetamol in patients with *Plasmodium falciparum* malaria [25], consisting of a two‐compartment disposition model with first‐order absorption describing the oral administration. This model demonstrated the lowest OFV, the most appropriate eta distributions with acceptable shrinkage, and external VPC performance that most adequately reflected the variability in the current population. Subsequently, three prior implementation approaches were evaluated, that is, informative prior on both θ and ω, informative prior on θ with non‐informative prior on ω, and informative prior on θ with ω estimated freely from the current data. Details on the prior model evaluation are provided in the Supplementary Files S2. The first‐order conditional estimation method with eta‐epsilon interaction (FOCE‐I) was used throughout model development. Model evaluations and diagnostics were carried out using the R‐package Xpose version 4.0, Perl‐speaks‐NONMEM version 4.8.0, and Pirana version 2.9.9 [28, 29, 30]. OFV were assumed to be *χ* ^2^ distributed and a drop of 3.84, 6.63 and 10.83 were deemed significant on a significance level of 0.05, 0.01 and 0.001, respectively. A total of 2.0% of paracetamol concentrations that measured below the lower limit of quantification (LLOQ) were omitted. For patients with measurable pre‐study concentrations, the compartment initialization method [31], in which the observed concentration at enrollment was used to initialize the amount of paracetamol in the central compartment, was implemented to handle the missing dose record prior to study enrollment. PK parameters were assumed to be log‐normally distributed, and inter‐individual variability (IIV) was implemented with an exponential function as shown in Equation (1).

(1) $$\theta_{i}=\theta \cdot e^{\eta_{\theta ,i}}$$

where $\theta_{i}$ denotes the individual parameter estimate, θ denotes the population mean parameter estimate, and $\eta_{\theta ,i}$ denotes the IIV with zero mean and variance $\omega^{2}$. Inter‐occasion variability (IOV), was also implemented with an exponential function as shown in Equation (2) to investigate the between days random variability, that is, day 0, day 1, day 2, and day 3, which was evaluated on the relative bioavailability parameter.

(2) $$\theta_{i,j}=\theta \cdot e^{\eta_{i,\theta }+\kappa_{j,\theta }}$$

where $\kappa_{i,\theta }$ denotes the IOV of the PK parameter with zero mean and variance $\omega^{2}$ at the *j*th occasion. The residual unexplained variability was assumed to be additive on a logarithmic scale. Additionally, a time‐dependent effect was evaluated for both the relative bioavailability and apparent elimination clearance of paracetamol to describe the reduction in observed peak concentrations at the last dose compared to the first dose (46% reduction; range: 12%–96%) despite constant dosing, which standard time‐invariant models failed to adequately describe. Exponential functions with a plateau constraint were evaluated as these naturally incorporate physiologically meaningful limits. The function used to describe the decrease in relative bioavailability over time is shown in Equation (3).

(3) $$\text{Time effect}\;\mathrm{on}\;\text{relative bioavailability}=e^{\left(-k\cdot t\right)}\times \left(1-\frac{F_{\mathrm{MIN}}}{F_{t=0}}\right)+\frac{F_{\mathrm{MIN}}}{F_{t=0}}$$

where *k* denotes the rate of bioavailability decrease over time, *t* denotes time in hours, *F*_*t* = 0_ denotes relative bioavailability at time zero (1 fixed) and *F*_MIN_ denotes the minimum relative bioavailability.

The function used to assess the time effect on the increase in apparent elimination clearance over time is shown in Equation (4).

(4) $$\text{Time effect}\;\mathrm{on}\;\text{clearance}=\left(1-e^{\left(-k\cdot t\right)}\right)\cdot \left(\frac{{\mathrm{CL}}_{\mathrm{MAX}}}{{\mathrm{CL}}_{t=0}}-1\right)+1$$

where *k* denotes the rate of apparent elimination clearance increase over time, CL_*t* = 0_ denotes apparent elimination clearance at time zero, *t* denotes time in hours, and CL_MAX_ denotes the maximum apparent elimination clearance.

The effect of body weight on PK parameters was evaluated using an allometric function as shown in Equation (5).

(5) $$\theta_{i}=\theta \cdot e^{\eta_{\theta ,i}}\cdot {\left(\frac{{\mathrm{BW}}_{i}}{{\mathrm{BW}}_{\text{median}}}\right)}^{n}$$

where ${\mathrm{BW}}_{i}$ denotes individual body weight and ${\mathrm{BW}}_{\text{median}}$ denotes median body weight of the study population and n was set to be equal to 0.75 and 1 for all clearance parameters and volume of distribution parameters, respectively. Physiologically relevant demographic covariates, including age, sex, baseline parasitemia, hemoglobin, hematocrit, aspartate aminotransferase, alanine aminotransferase (ALT), and severe malaria were investigated using a stepwise covariate approach (*p* values of < 0.05 and < 0.01 for forward inclusion and backward elimination, respectively). Model diagnostics, including goodness‐of‐fit plots and prediction‐corrected visual predictive checks [32] (*n* = 1000), were used to assess the descriptive and predictive performance of the model, respectively. The uncertainty and robustness of the PK parameters estimated from the final model were assessed using bootstrapping (*n* = 1000).

### 2.3 Population Pharmacokinetic/Pharmacodynamic Modeling of Paracetamol

For the PK/PD analysis of paracetamol, the individual PK parameters derived from the developed PK model of paracetamol were used to generate individual concentration‐time profiles, which were then linked to the PD parameters of interest. In the current analysis, the PD parameters that were evaluated include creatinine, fever clearance time, ALT, and parasite clearance. Model diagnostics and parameter uncertainty were evaluated in the same manner as described above for the PK model.

#### 2.3.1 Effect of Paracetamol on Creatinine

Model structure for percentage change in creatinine from baseline was based on visual inspection and physiological plausibility. Creatinine profiles showed a rapid initial decline followed by a plateau over 72 h. Both linear and exponential plateau models were evaluated to describe the natural change of creatinine from baseline, as shown in Equations (6) and (7), respectively.

(6) $$∆\text{Creatinine}\;\left(\%\right)={\text{Base}}_{\mathrm{CR}}+{\text{Slope}}_{\mathrm{CR}}\cdot t$$

(7) $$\begin{array}{c} ∆\text{Creatinine}\;\left(\%\right)=\left({\text{Base}}_{\mathrm{CR}}-\text{Plateau}\right)\cdot e^{-\kappa t}+\text{Plateau} \end{array}$$

where ∆Creatinine denotes percentage change of creatinine from baseline, Base_CR_ denotes the ∆Creatinine at time 0 (fixed to zero), Slope_CR_ denotes the linear slope of percentage change of creatinine over time, κ denotes the rate constant of creatinine reduction, *t* denotes time in hours, and Plateau denotes the maximum percentage change of creatinine from baseline. The effect of paracetamol was then evaluated on the model that best described the natural change of creatinine, including treatment arm, AUC_0‐72H_, and paracetamol concentration. For effect of paracetamol concentration, both linear and *E*_MAX_ functions, with either additive or proportional effects, were evaluated on the parameters describing the percentage change of creatinine over time (i.e., Slope_CR_, *κ*, and Plateau). The linear and *E*_MAX_ concentration‐effect models were described using Equations (8) and (9), respectively.

(8) $$\mathrm{EFF}={\text{Slope}}_{\mathrm{CP}}\cdot C_{P}$$

(9) $$\mathrm{EFF}=\frac{E_{\mathrm{MAX}}\cdot C_{P}}{{\mathrm{EC}}_{50}+C_{P}}$$

where EFF denotes the concentration‐effect of paracetamol, ${\text{Slope}}_{\mathrm{CP}}$ denotes the slope of concentration‐effect relationship, *E*_MAX_ denotes the maximum effect, EC_50_ denotes paracetamol concentration at which effect is 50% of the *E*_MAX_, and *C*_*P*_ denotes paracetamol plasma drug concentration. Physiologically plausible covariates including, KDIGO stage at enrollment, severe malaria, cell‐free hemoglobin ≥ 77,600 ng/mL, and patient developed acute kidney injury after enrollment were evaluated using stepwise covariate approach.

#### 2.3.2 Effect of Paracetamol on Fever and Parasite Clearance Time

Fever clearance time was analyzed using a parametric time‐to‐event modeling approach. Two endpoints were assessed: fever clearance time A (FCT‐A; time to first temperature < 37.5°C) and fever clearance time B (FCT‐B; time to temperature < 37.5°C and remain there for 24 h). Data from the paracetamol and control groups were modeled simultaneously in NONMEM using Laplacian estimation. Exponential, Gompertz, Weibull, and log‐logistic hazard functions were evaluated, and the best‐fitting model was selected. Paracetamol effects were evaluated on the hazard function as described previously, using treatment arm, AUC_0‐72H_, and plasma concentrations, with linear and *E*_MAX_ relationships explored. Additional covariates including severe malaria and cell‐free hemoglobin ≥ 77,600 ng/mL were also evaluated using a stepwise covariate approach. Full model details are provided in the Supplementary Files S3.

In addition, parasite clearance was explored following a previous analysis [20] that showed slightly increased parasite clearance parameters in the paracetamol group. Associations between parasite clearance parameters (slope half‐life, PC_50_–PC_99_) and paracetamol exposure (*C*_MAX_, AUC_0‐72H_) were evaluated using linear regression in GraphPad Prism.

#### 2.3.3 Effect of Paracetamol on Liver Function

The measurements of ALT were transformed to their natural logarithms. The log‐linear function as shown in Equation (10) was used to describe the baseline change in ALT level over time, starting from admission until day 7 (0–168 h).

(10) $$\ln\left(\mathrm{ALT}\right)={\text{Base}}_{\ln\left(\mathrm{ALT}\right)}+{\text{Slope}}_{\ln\left(\mathrm{ALT}\right)}\cdot t$$

where ln(ALT) denotes the natural logarithm of ALT level, Base_ln(ALT)_ denotes the baseline natural logarithm ALT level at time 0, Slope_ln(ALT)_ denotes the linear slope of ln(ALT) change over time, and t denotes time in hours. Paracetamol effects were evaluated on the slope of ln(ALT) change as described previously, using treatment arm, AUC_0‐72H_, and plasma concentrations, with linear and *E*_MAX_ relationships explored. Additional covariates including baseline serum creatinine, baseline ALT level, age, body weight, KDIGO stage at enrollment, severe malaria, cell‐free hemoglobin ≥ 77,600 ng/mL, and sex were evaluated on both baseline and slope of the log‐linear function using a stepwise covariate approach.

## 3 Results

A total of 372 patients from the PACKNOW study (paracetamol: *n* = 183, control: *n* = 189) were included in the PK/PD analyses (Table 1). Five patients who had undergone RRT and one patient who had incomplete data were excluded from the PK/PD analysis (Figure S1). The study cohort included 28 (7.5%) patients with WHO‐defined severe malaria on research criteria, in addition to 106 (28.5%) with AKI (defined as KDIGO stage 0–3) [23].

**TABLE 1:** Demographics and baseline characteristics of patients by treatment group.

Characteristics	Paracetamol (*n* = 183)	Control (*n* = 189)
Age (years)	35 (26–49)	36 (24–48)
Male sex, *n* (%)	160 (87.4)	154 (81.5)
Weight (kg)	61.0 (53.5–68.4)	58.0 (52.0–66.5)
Temperature (°C)	37.5 (37.0–38.2)	37.4 (37.0–38.0)
Complications at enrollment		
Parasitemia ≥ 20,000/μL, *n* (%)	18 (9.8)	18 (9.5)
Severe malaria, *n* (%)	13 (7.1)	15 (7.9)
Renal dysfunction, *n* (%)		
KDIGO stage 0	134 (73.2)	132 (69.8)
KDIGO stage 1	39 (21.3)	52 (27.5)
KDIGO stage 2	8 (4.4)	5 (2.6)
KDIGO stage 3	2 (0.6)	—
All KDIGO stages (1–3)	49 (26.8)	57 (30.2)
Laboratory investigations		
Parasite count (parasites/μL)	1716 (558–7287)	2636 (640–8740)
Creatinine (μmol/L)	83 (54–68)	84 (74–99)
Cell‐free hemoglobin (ng/mL)	30,872 (16,085–63,576)	25,799 (13,183–56,245)
Hemoglobin (g/dL)	13.9 (12.6–15.0)	13.8 (12.2–14.7)
Aspartate aminotransferase (U/L)	28 (19–42)	29 (20–41)
Alanine aminotransferase (U/L)	35 (23–55)	33 (22–48)
Paracetamol sampling schedule		
Dense sampling, *n* (%)	22 (12.0)	—
Sparse sampling, *n* (%)	161 (88.0)	189 (100.0)

*Note:* Values are presented as median (interquartile range) unless otherwise specified. Baseline was defined as the enrollment measurements prior to study drug administration.

Abbreviation: KDIGO, Kidney Diseases: Improving Global outcomes.

### 3.1 Population Pharmacokinetics of Paracetamol

**FIGURE 1:**
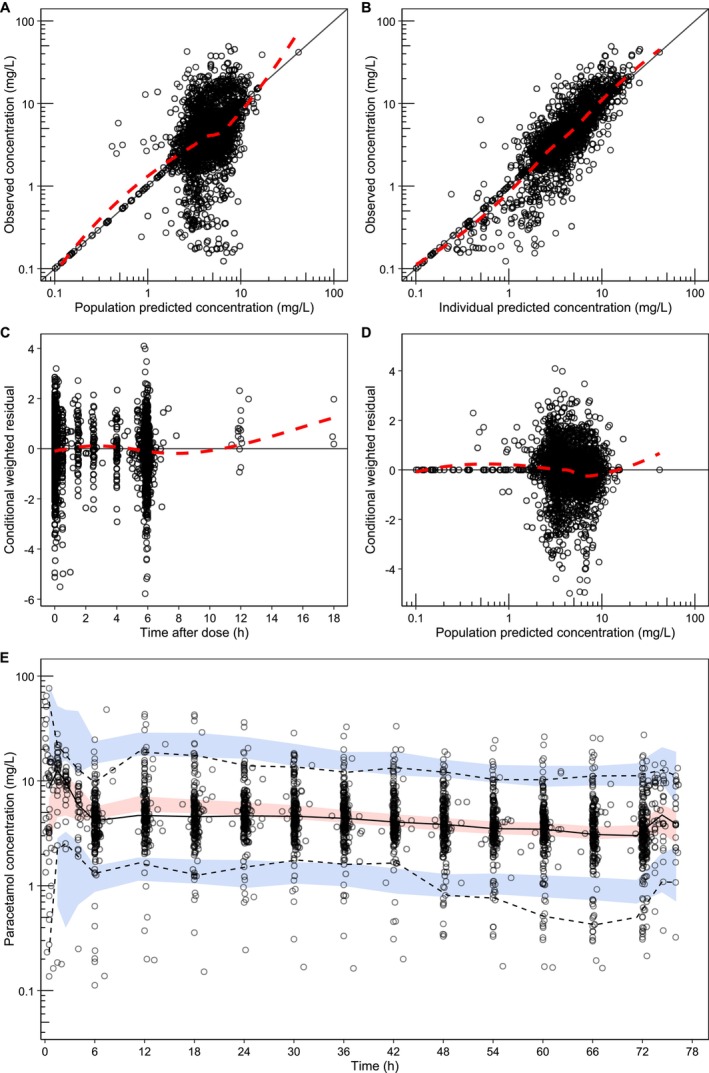
Goodness‐of‐fit plots and prediction‐corrected visual predictive check of the final population pharmacokinetic model of paracetamol in *Plasmodium knowlesi* patients. Observed concentrations versus population predictions (A), observed concentrations versus individually predicted concentrations (B), conditionally weighted residuals vs. time after dose (C), and conditionally weighted residuals versus population predictions (D). The open circles represent the observed concentrations. The solid black lines represent the line of identity or zero‐line, and the dashed red lines represent a local polynomial regression fitting of all data (trend lines). Prediction‐corrected visual predictive check (*n* = 1000) of the final population pharmacokinetic model of paracetamol (E). The open circles represent the observed paracetamol concentrations. The solid black line represents the 50th percentile of the observations, and dashed black lines represent the 5th and 95th percentiles of the observations. The shaded areas represent the 95% confidence intervals of each simulated percentile.

A total of 2435 paracetamol plasma samples from 183 patients were analyzed, comprising 453 (18.6%) intensive and 1982 (81.4%) sparse samples. Incorporating prior information from a previously published model in *Plasmodium falciparum* patients [25], using an informative prior on θ while allowing ω to be estimated freely from the current dataset, helped stabilize parameter estimation and resulted in the best model fit. Paracetamol PK were best described by a two‐compartment model with first‐order absorption and elimination. Inter‐occasion variability on relative bioavailability accounted for differences between dosing occasions (*p* < 0.001). Bioavailability decreased significantly over time (*p* < 0.001), reaching a minimum relative bioavailability of 0.220 (95% CI: 0.145–0.301), with a decline half‐life of 10.2 h (95% CI: 6.70–16.6). The impact of body weight on clearance and volume of distribution parameters was described by an allometric function, where an increase in body weight led to higher paracetamol clearance and volume of distribution. Increasing age was associated with slower absorption, with the absorption rate constant decreasing by 1.9% per year (95% CI: 1.6–2.0; *p* < 0.01). The population PK parameter estimates were robust, with relative standard errors below 30% (Table 2), except for IIV on absorption rate constant (48.7%) and on the apparent volume of distribution of peripheral compartment (39.0%). The goodness‐of‐fit plots and the visual predictive check for the final model demonstrated good descriptive and predictive performance (Figure 1).

**TABLE 2:** Population pharmacokinetic parameters of paracetamol in Malaysian patients with *P. knowlesi* malaria.

Parameters	Population estimates^a^ (%RSE)^b^	95% CI^b^ [% Shrinkage]^a^
Fixed effects		
*F*	1 Fixed	—
*k*_*a*_ (h^−1^)	0.917 (18.3)	0.538–1.20
CL/*F* (L/h)	9.80 (11.2)	7.74–12.3
*V*_*C*_/*F* (L)	53.7 (4.1)	48.6–57.9
*Q*/*F* (L/h)	10.7 (15.2)	7.92–14.7
*V*_*P*_/*F* (L)	33.2 (7.1)	27.8–38.2
$\;\theta_{\mathrm{AGE}}$on *k*_*a*_	−0.0187 (8.1)	−0.0198 to −0.0160
Time‐dependent effect on *F*		
*F*_50_ (h)	10.2 (23.5)	6.70–16.6
*F*_MIN_	0.220 (16.6)	0.145–0.301
Random effects (%CV)		
$\;{\mathrm{IIV}}_{F}$	26.2 (25.8)	9.0–38.2 [47.4]
$\;{\mathrm{IIV}}_{k_{a}}$	272 (48.7)	131–572 [34.4]
${\mathrm{IIV}}_{V_{C}}$	63.4 (21.3)	28.3–122 [50.4]
$\;{\mathrm{IIV}}_{V_{P}}$	387 (39.0)	204–793 [41.3]
$\;{\mathrm{IOV}}_{F}$	53.4 (13.7)	40.3–69.1[38.1]
*σ*	0.290 (8.3)	0.246–0.340 [11.0]

Secondary pharmacokinetic parameters of paracetamol		
Parameters	Median	Interquartile range
First dose *C*_MAX_ (mg/L)	9.34	6.79–13.6
First dose *T*_MAX_ (h)	1.51	1.13–2.10
Last dose *C*_MAX_ (mg/L)	4.15	2.91–5.57
Last dose *T*_MAX_ (h)	1.21	0.697–1.50
AUC_0‐72H_ (mg × h/L)	408	331–504
Half‐life (h)	9.31	7.07–12.8

Abbreviations: $\theta_{\mathrm{AGE}}$, effect of age on *k*_*a*_; *σ*, variance of the residual variability incorporated as an additive error on the logarithmic scale; AUC_0‐72H_, area under the concentration‐time curve from time 0 to 72 h.CL/F, apparent elimination clearance; *C*_MAX_, maximum concentration; *F*, relative bioavailability; *F*_50_, time at which the relative bioavailability was reduced by 50%; *F*_MIN_, minimum relative bioavailability; *k*_*a*_, first‐order absorption rate constant; *Q*/*F*, apparent intercompartmental clearance; *T*_MAX_, time to reach maximum concentration; *V*_*C*_/*F*, apparent volume of distribution of the central compartment; *V*_*P*_/*F*, apparent volume of distribution of peripheral compartment.

^a^ Population mean values, inter‐individual variability (IIV) and inter‐occasion variability (IOV) were estimated by NONMEM.

^b^ Relative standard error (% RSE) and 95% confidence interval (95% CI) were assessed by 1000 bootstrap runs. Coefficient of variation (% CV) for IIV and IOV was calculated as $100\times \sqrt{\exp\left(\text{estimate}\right)–1}$.

### 3.2 Population Pharmacokinetics/Pharmacodynamics of Paracetamol

#### 3.2.1 Effect of Paracetamol on Creatinine

A total of 2365 creatinine measurements from 372 patients in the paracetamol (*n* = 183) and control (*n* = 189) groups were analyzed. Samples were collected at enrolment (baseline) and every 12 h up to 72 h after commencement of paracetamol administration. The percentage change from baseline creatinine was calculated for each patient at each time point and included in the PK/PD analysis. The data were best described by a model incorporating an exponential decay with a plateau function to represent the percentage change from baseline. Higher paracetamol exposure was associated with a faster creatinine reduction rate (*p* < 0.01). The reduction rate increased by 25.4% (95% CI: 15.5–28.8) for every 10‐fold increase in paracetamol AUC_0‐72H_, with a mean reduction rate constant of 0.0477 h^−1^ (95% CI: 0.0386–0.0502). The mean maximal reduction (plateau) in creatinine was estimated at 9.9% (95% CI: 7.9–11.2) below baseline. The severity of AKI at enrollment, based on ordinal KDIGO staging of 0 to 3, significantly influenced the maximal proportional change in creatinine plateau (*p* < 0.001), with greater improvements observed in patients with higher KDIGO stages at enrollment.

**FIGURE 2:**
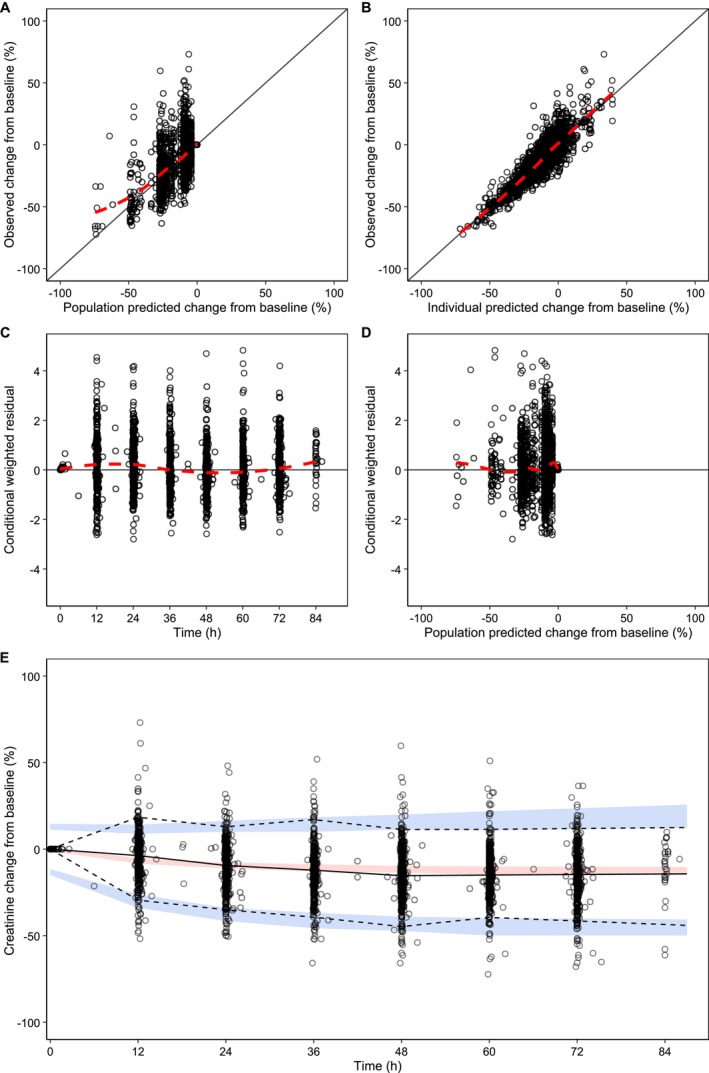
Goodness‐of‐fit plots and visual predictive check of the final PK/PD model for paracetamol effect on creatinine. Observed creatinine change from baseline versus population prediction (A), observed creatinine change from baseline versus individual prediction (B), conditionally weighted residuals versus time (C), and conditionally weighted residuals versus population prediction (D). The open circles represent the observed creatinine change from baseline. The solid black lines represent the line of identity or zero‐line, and the dashed red lines represent a local polynomial regression fitting of all data (trend lines). Visual predictive check (*n* = 1000) of the final PK/PD model for paracetamol effect on creatinine (E). The open circles represent the observed creatinine change from baseline. The solid black line represents the 50th percentile of the observations, and the dashed black lines represent the 5th and 95th percentiles of the observations. The shaded areas represent the 95% confidence intervals of each simulated percentile.

Subset analyses of severe and non‐severe *Plasmodium knowlesi* malaria showed a similar trend, with paracetamol exposure increasing the rate of creatinine reduction in both groups. For each 10‐fold increase in paracetamol AUC_0‐72H_, the reduction rate increased by 26.1% (95% CI: 8.2–44.0; *p* < 0.01) in non‐severe malaria and by 23.2% (95% CI: −30.3 to 64.9; *p* > 0.05) in severe malaria. The corresponding mean rate constants were 0.0507 (95% CI: 0.0394–0.0620) and 0.0349 (95% CI: 0.00230–0.0686), respectively. The lack of statistical significance in the severe malaria group may be due to the smaller sample size in this cohort. The parameter estimates from the final PK/PD model describing the percentage change from baseline of creatinine for the entire cohort were robust, with relative standard errors below 30% (Table 3). The goodness‐of‐fit plots and the visual predictive check for the final model demonstrated good descriptive and predictive performance (Figure 2).

**TABLE 3:** Parameter estimates from the population pharmacokinetic/pharmacodynamic parameters describing the percentage change from baseline of serum creatinine.

Parameters	Population estimates^a^ (%RSE)^b^	95% CI^b^ [% Shrinkage]^a^
Fixed effects		
κ (h^−1^)	0.0477 (5.6)	0.0386–0.0502
Plateau (%)	−9.9 (8.0)	−11.2 to −7.9
$\;\theta_{\mathrm{AUC}72H}\;$ on κ	0.254 (12.9)	0.155–0.288
$\;\theta_{\text{KDIGO}}$ on Plateau		
$\theta_{\text{KDIGO}-1}$	1.77 (8.9)	1.56–2.24
$\theta_{\text{KDIGO}-2}$	3.93 (8.5)	3.88–5.00
$\theta_{\text{KDIGO}-3}$	6.52 (19.2)	6.11–7.83
Random effects^c^		
$\;{\mathrm{IIV}}_{\kappa }$	0.0377 (9.8)	0.0319–0.0407 [43.8]
$\;{\mathrm{IIV}}_{\text{Plateau}}$	12.2 (10.0)	10.1–12.5 [15.7]
*σ*	7.94 (5.7)	7.46–8.46 [9.4]

Abbreviations: $\theta_{\text{KDIGO}}$, effect of KDIGO stage on Plateau; $\theta_{\text{KDIGO}-1}$, effect of KDIGO stage 1; $\theta_{\text{KDIGO}-2}$, effect of KDIGO stage 2; $\theta_{\text{KDIGO}-3}$, effect of KDIGO stage 3; $\theta_{\mathrm{AUC}72H}$, effect of paracetamol exposure (log_10_AUC_0‐72H_) on κ; *σ*, additive error; κ, rate constant of exponential decay.

^a^ Population mean values and inter‐individual variability (IIV, additive) were estimated by NONMEM.

^b^ Relative standard error (% RSE) and 95% confidence interval (95% CI) were assessed by 1000 bootstrap runs.

^c^ Random effects were reported on the arithmetic scale, as they were implemented in the model using an additive function.

#### 3.2.2 Effect of Paracetamol on Fever and Parasite Clearance Time

In the PACKNOW trial, the median FCT‐B was significantly shorter in the paracetamol arm compared to the control arm [20]. In this PK/PD analysis, we further evaluated the effect of paracetamol exposure on fever clearance time using the time‐to‐event model. Among patients included in the PK/PD analysis, 195 patients had a temperature of ≥ 37.5°C at enrollment. One patient in the control arm with missing data was excluded, leaving 194 patients (102 in paracetamol and 92 in the control group) for analysis.

For both FCT‐A and FCT‐B, a log‐logistic hazard model best described the data, and was further improved by incorporating paracetamol exposure (AUC_0‐72H_) as a linear covariate on the hazard function (*p* < 0.01). For FCT‐A, higher paracetamol exposure was associated with an approximately 15.8% (95% CI: 4.0–28.0) faster rate of fever clearance per 100 mg·h/L increase in AUC_0‐72H_. For FCT‐B, a similar association was observed, with an approximately 12.6% (95% CI: 2.0–25.0) faster rate of sustained fever clearance per 100 mg·h/L increase in AUC_0‐72H_. Parameter estimates from the time‐to‐event models were robust, with relative standard errors below 30%, except for the paracetamol exposure effects on FCT‐A (45.7%) and FCT‐B (50.0%) (Table S1). The visual predictive checks demonstrated that the models adequately described and predicted the observed fever clearance profiles (Figure S2).

Linear regression of paracetamol exposure (C_MAX_ and AUC_0‐72H_) against parasite clearance parameters (slope half‐life, PC_50_–PC_99_) showed no significant associations (*p* > 0.05; Figure S3).

#### 3.2.3 Effect of Paracetamol on Liver Function

A total of 372 patients in the paracetamol (*n* = 183) and control (*n* = 189) groups were included in this analysis. Changes in ALT levels, used as a biomarker of hepatotoxicity, were evaluated over 0–168 h and were best described by a log‐linear model. Incorporating paracetamol exposure (AUC_0‐72H_) as a covariate on the slope of ALT change significantly improved the model (*p* < 0.001). Stepwise covariate analysis identified age (*p* < 0.01), baseline ALT (*p* < 0.001), and body weight (*p* < 0.001) as additional significant covariates compared with the model that included the impact of paracetamol exposure.

**FIGURE 3:**
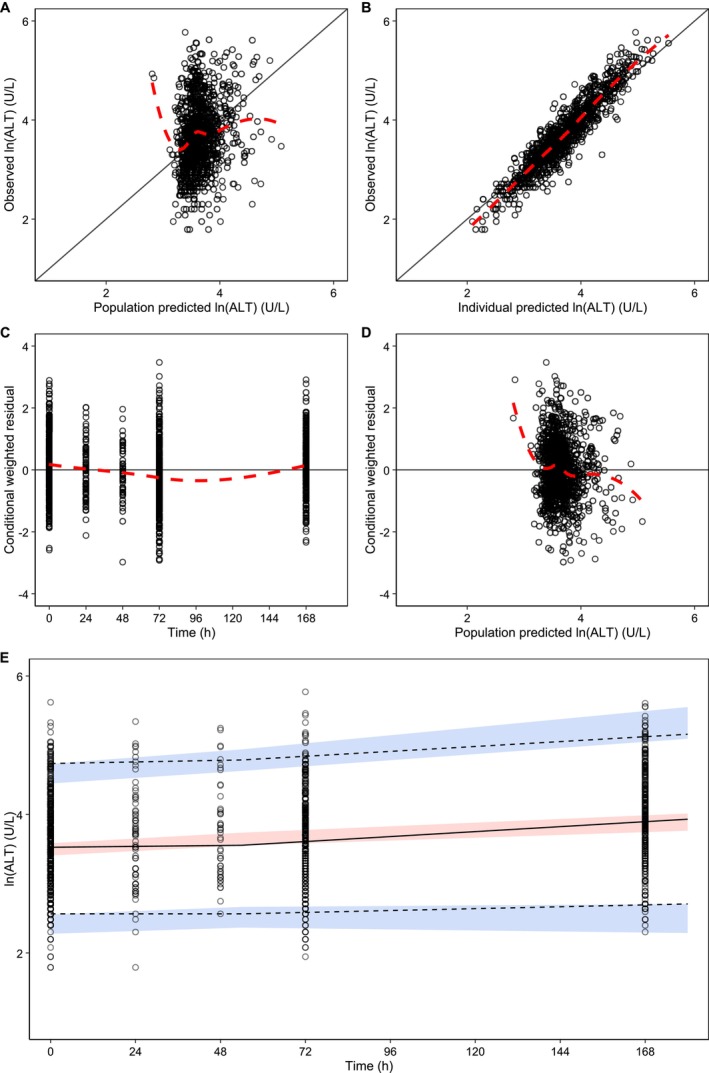
Goodness‐of‐fit plots and visual predictive check of PK/PD model for paracetamol effect on alanine aminotransferase (ALT). Observed ln (ALT) versus population prediction (A), observed ln (ALT) versus individual prediction (B), conditionally weighted residuals versus time (C), and conditionally weighted residuals versus population prediction (D). The open circles represent the observed ln (ALT). The solid black lines represent the line of identity or zero‐line, and the dashed red lines represent a local polynomial regression fitting of all data (trend lines). Visual predictive (*n* = 1000) of the final PK/PD model for paracetamol effect on ALT (E). The open circles represent the observed ln (ALT). The solid black line represents the 50th percentile of the observations, and the dashed black lines represent the 5th and 95th percentiles of the observations. The shaded areas represent the 95% confidence intervals of each simulated percentile. ln, natural logarithm.

Age and baseline ALT were inversely associated with the slope of ALT change, whereas body weight was positively associated with baseline ALT. In the final model, paracetamol exposure increased the ALT slope by 0.3% (95% CI: 0.2–0.5) per 1 mg · h/L increase in AUC_0‐72H_. Each additional year of age reduced the slope by 1.1% (95% CI: 0.3–2.0), and each 1 U/L increase in baseline ALT reduced the slope by 1.2% (95% CI: 0.8–1.7). Baseline ALT increased by 0.4% (95% CI: 0.2–0.5) for every 1 kg increase in body weight. From this model, although increases in paracetamol exposure were associated with increases in ALT, within the observed AUC_0‐72H_ range in the study population receiving paracetamol every 6 h for 72 h, none of the individual predicted ALT values at any time point exceeded three times their baseline (range: −0.965 to 1.71). The parameter estimates from the final model were robust, with relative standard errors below 30% for all parameters, except for the effect of age on the slope (36.1%) (Table 4). The goodness‐of‐fit plots and the visual predictive checks demonstrated a good descriptive and predictive performance of the model (Figure 3).

**TABLE 4:** Parameter estimates from the population pharmacokinetic/pharmacodynamic parameters describing the alanine aminotransferase change over time.

Parameters	Population estimates^a^ (%RSE)^b^	95% CI^b^ [% Shrinkage]^a^
Fixed effects		
Slope (h^−1^)	0.00236 (11.4)	0.00185–0.00289
Intercept (U/L)	3.49 (0.9)	3.43–3.56
$\;\theta_{\mathrm{AGE}}$ on slope (% per year)	−1.1 (36.1)	−2.0 to −0.3
$\;\theta_{{\mathrm{AUC}}_{0-72H}}\;$ on slope (% per mg · h/L)	0.3 (26.0)	0.2–0.5
$\;\theta_{\text{BASEALT}}$ on slope (% per U/L)	−1.2 (19.2)	−1.7 to −0.8
$\;\theta_{\mathrm{WT}}\;$on intercept (% per kg)	0.4 (23.6)	0.2–0.5
Random effects		
$\;{\mathrm{IIV}}_{\text{Slope}}$	0.00274 (10.2)	0.00213–0.00320 [37.9]
$\;{\mathrm{IIV}}_{\text{Intercept}}$	0.547 (5.0)	0.496–0.597 [9.2]
*σ*	0.118 (10.3)	0.0965–0.142 [24.1]

Abbreviations: $\theta_{{\mathrm{AUC}}_{0-72H}}$, effect of area under the concentration‐time curve from time 0 to 72 h on slope; $\theta_{\mathrm{AGE}}$, effect of age on the slope; $\theta_{\text{BASEALT}}$, effect of baseline alanine aminotransferase on slope; $\theta_{\mathrm{WT}}$, effect of body weight on intercept; *σ*, additive error.

^a^ Population mean values and inter‐individual variability (IIV, additive) were estimated by NONMEM.

^b^ Relative standard error (% RSE) and 95% confidence interval (95% CI) were assessed by 1000 bootstrap runs.

## 4 Discussion

In this study, we described the population PK/PD of paracetamol in Malaysian patients with *Plasmodium knowlesi* malaria, utilizing data from the PACKNOW clinical trial [20] to evaluate the impact of paracetamol exposure on renal, hepatic, and fever outcomes. The PK/PD model for creatinine showed that higher paracetamol exposure significantly accelerated the decline in creatinine following treatment, supporting the renoprotective effects of paracetamol observed in PACKNOW. Importantly, in the PACKNOW study, a renoprotective effect was demonstrated in subgroups of patients with severe knowlesi malaria, and with AKI and hemolysis, but not in the overall cohort. However, our PK/PD paracetamol findings extend these observations by suggesting potential benefit even in patients with uncomplicated *Plasmodium knowlesi* malaria, who comprised the majority of patients in this analysis. These results are also consistent with our previous study in *Plasmodium falciparum* malaria, where higher paracetamol exposure (AUC_0‐72H_) was associated with improved renal outcomes [19]. Together, these data provide evidence for an exposure‐dependent renoprotective effect of paracetamol in malaria.

In addition to improving renal function, higher paracetamol exposure was also associated with faster fever clearance times, confirming the antipyretic effect of paracetamol in patients with *Plasmodium knowlesi* malaria. These findings are consistent with previous studies in *Plasmodium falciparum* malaria [19, 20, 33, 34, 35, 36] and support current WHO recommendations for paracetamol use as an antipyretic [37]. Concerns raised by earlier studies about a lack of antipyretic benefit may reflect underdosing, infrequent body temperature assessments, or sub‐therapeutic paracetamol concentrations, as drug levels were not measured in those analyses [38, 39].

Paracetamol exposure was also associated with elevations in ALT. Our model identified that higher paracetamol exposure increased the slope of ALT rise over time, while age and baseline ALT attenuated this effect. These relationships are consistent with findings from larger observational studies [40]. Importantly, although an elevation in predicted ALT was observed with higher paracetamol exposure, for all participants, these values were well below Hy's law criteria for hepatotoxicity, based on an elevation three times greater than the measured baseline, and no significant differences in adverse events were noted between the treatment and control groups. Together with safety data from a previous study in falciparum malaria [19], these findings support the tolerability of standard‐dose paracetamol in malaria.

In this study, a PK/PD analysis of paracetamol demonstrated no significant relationship between paracetamol exposure and parasite clearance time. This finding aligns with previous PK/PD of paracetamol and randomized trial data in falciparum and knowlesi malaria [19, 20, 25, 41]. Although earlier studies, including a Cochrane review and a trial in African children, reported inconclusive or conflicting results regarding parasite clearance time [38, 42], the current analysis supports that while paracetamol provides renoprotective and antipyretic benefits, it does not influence parasite clearance dynamics.

The PK analysis of paracetamol in this study demonstrated that the exposure to paracetamol declined over time, despite continued dosing. The current data are insufficient to differentiate whether the time dependency is attributable to changes in relative bioavailability or elimination clearance of the drug, as both similarly affect paracetamol exposure. In our model, implementing the time effect on bioavailability provided a better fit and was therefore retained. The decline in paracetamol exposure over time may be related to altered drug metabolism during acute malaria infection. Paracetamol is primarily metabolized via glucuronidation and sulfation, with a minor oxidative pathway generating toxic metabolite N‐acetyl‐p‐benzoquinone imine (NAPQI) [43]. In malaria, glucuronidation has been shown to decrease, potentially due to reduced availability of UDP‐glucuronic acid during acute illness [44, 45]. Animal studies have also demonstrated downregulation of UGT, SULT, and CYP enzyme expression during peak parasitemia, with recovery thereafter [46]. Although these mechanistic findings align with our observations, this study did not directly assess paracetamol metabolism to determine whether reduced metabolism contributed to the decline in paracetamol exposure over time. A pooled analysis of PK studies of paracetamol in malaria patients, incorporating metabolite measurements, could help elucidate the mechanisms underlying this phenomenon.

In conclusion, this PK/PD analysis of paracetamol demonstrates clear exposure–response relationships between paracetamol and clinical outcomes in *Plasmodium knowlesi* malaria. Higher exposure was associated with improved renal function and faster fever clearance, while also linked to modest increases in ALT without evidence of hepatotoxicity. These findings support the renoprotective and antipyretic effects of paracetamol in malaria and support a wider uptake of its use for these purposes in severe malaria.

## Author Contributions

Thanaporn Wattanakul, Richard M. Hoglund, and Katherine Plewes wrote the manuscript. Daniel J. Cooper, Katherine Plewes, Matthew J. Grigg, Giri S. Rajahram, Timothy William, Arjen M. Dondorp, Michael D. Edstein, Geoffrey W. Birrell, Nicholas M. Anstey, and Bridget E. Barber designed and performed the research. Thanaporn Wattanakul, Richard M. Hoglund, and Joel Tarning analyzed the data. All authors revised and approved the final manuscript.

## Funding

This work was supported by Ministry of Health, Malaysia (grant number BP00500420 and grant number BP00500/117/1002 to Giri S. Rajahram); the US NIH (5R01_AI160457‐02 to Giri S. Rajahram and Matthew J. Grigg); the Australian National Health and Medical Research Council (grants 1037304, 1132975, and 1045156 and fellowships to Nicholas M. Anstey, 1042072, 1135820; Bridget E. Barber, 1088738; and Matthew J. Grigg 2017436); the Australian Centre of Research Excellence in Malaria Elimination (ACREME); and the Wellcome Trust (grant 220211). The authors used AI assistance for language editing and improving the readability of this manuscript. This research was funded in part, by the Wellcome Trust [315982/Z/24/Z]. For the purpose of Open Access, the author has applied a CC BY public copyright license to any Author Accepted Manuscript version arising from this submission.

## Disclosure

Disclaimer: The views expressed here are those of the authors and do not necessarily reflect the official policy or position of the Australian Defense Force, Joint Health Command, or any extant Australian Defense Force policy.

## Conflicts of Interest

The authors declare no conflicts of interest.

## Supporting information


**Figure S1:** Participant flow diagram. PACKNOW, clinical trial entitled—effect of regularly dosed paracetamol versus no paracetamol on renal function in plasmodium knowlesi Malaria; PCR, polymerase chain reaction, PK analysis, pharmacokinetic analysis; PK/PD analysis, pharmacokinetic/pharmacodynamic analysis.
**Figure S2:** Visual predictive checks (*n* = 1000) of the final time‐to‐event model describing paracetamol effect on fever clearance time. Visual predictive checks (*n* = 1000) of the final time‐to event model describing FCT‐A (A) and FCT‐B (B). FCT‐A, time taken for the temperature to fall below 37.5°C; FCT‐B, time taken for the temperature to fall below 37.5°C and remain there for at least 24 h.
**Figure S3:** Relationship between paracetamol exposure and parasite clearance parameters. Slope half‐life versus C_MAX_ (A), PC_50_ versus C_MAX_ (B), PC_90_ versus C_MAX_ (C), PC_95_ versus C_MAX_ (D), PC_99_ versus C_MAX_ (E), Slope half‐life versus AUC_0‐72H_ (F), PC_50_ versus AUC_0‐72H_ (G), PC_50_ versus AUC_0‐72H_ (H), PC_50_ versus AUC_0‐72H_ (I), and PC_50_ versus AUC_0‐72H_ (J). None of the slopes of the linear regression models were significantly different from zero (*p* value > 0.05). The open circles represent the observations, black solid line represent the slope of the linear regression, and the shaded are represent the 95% confidence interval of the slope.
**Table S1:** Parameter estimates from the time‐to‐event models for fever clearance time.
