# Supplementary material for: Population Pharmacokinetics and Pharmacodynamics of Paracetamol in Malaysian Patients With Plasmodium knowlesi Malaria
Source: CPT Pharmacometrics Syst Pharmacol. 2026 Jun 22;15(7):e70283. doi: 10.1002/psp4.70283 (PMC13284899; doi:10.1002/psp4.70283)
Supplement: Supplementary file 1 — Figure S1: Participant flow diagram. PACKNOW, clinical trial entitled—effect of regularly dosed paracetamol versus no paracetamol on renal function in plasmodium knowlesi Malaria; PCR, polymerase chain reaction, PK analysis, pharmacokinetic analysis; PK/PD analysis, pharmacokinetic/pharmacodynamic analysis. Figure S2: Visual predictive checks (n = 1000) of the final time‐to‐event model describing paracetamol effect on fever clearance time. Visual predictive checks (n = 1000) of the final time‐to event model describing FCT‐A (A) and FCT‐B (B). FCT‐A, time taken for the temperature to fall below 37.5°C; FCT‐B, time taken for the temperature to fall below 37.5°C and remain there for at least 24 h. Figure S3: Relationship between paracetamol exposure and parasite clearance parameters. Slope half‐life versus CMAX (A), PC50 versus CMAX (B), PC90 versus CMAX (C), PC95 versus CMAX (D), PC99 versus CMAX (E), Slope half‐life versus AUC0‐72H (F), PC50 versus AUC0‐72H (G), PC50 versus AUC0‐72H (H), PC50 versus AUC0‐72H (I), and PC50 versus AUC0‐72H (J). None of the slopes of the linear regression models were significantly different from zero (p value > 0.05). The open circles represent the observations, black solid line represent the slope of the linear regression, and the shaded are represent the 95% confidence interval of the slope. Table S1: Parameter estimates from the time‐to‐event models for fever clearance time. [file PSP4-15-e70283-s001.pdf]

Supplementary material

Population Pharmacokinetics and Pharmacodynamics of Paracetamol in Malaysian Patients with *Plasmodium knowlesi* Malaria

Thanaporn Wattanakul, Daniel J Cooper, Katherine Plewes, Matthew J Grigg, Giri S Rajahram, Timothy William, Arjen M Dondorp, Michael D Edstein, Geoffrey W Birrell, Nicholas M Anstey, Richard M Hoglund, Bridget E Barber, Joel Tarning

---

S1. Complete detail of the study procedures

This pharmacokinetic/pharmacodynamic (PK/PD) analysis of paracetamol was pre-specified within the PACKNOW clinical trial entitled: Effect of Regularly Dosed Paracetamol vs No Paracetamol on Renal Function in *Plasmodium knowlesi* Malaria. The PACKNOW trial was a two-arm, open-label, randomized, controlled study conducted at one tertiary referral hospital and three district hospitals in Sabah, Malaysia. Eligibility included: age ≥ 5 years, hospitalized with microscopy-diagnosed knowlesi malaria, temperature exceeding 38°C or had a fever within the previous 48 hours, were within 18 hours of initiating antimalarial treatment with artesunate and/or artemether-lumefantrine, and provided written informed consent.

Exclusion criteria included: pregnancy, contraindication or allergy to paracetamol, known cirrhosis, or consumption of more than six standard alcoholic drinks per day. Patients without confirmed *Plasmodium knowlesi* monoinfection by polymerase chain reaction were excluded retrospectively. Patients fulfilling WHO research criteria for severe knowlesi malaria were classified as severe malaria, and the remainder as non-severe malaria. The study received approval from the Malaysian Research Ethics Committee (protocol number NMRR-16-356-29088) and the Ethics Committee of Menzies School of Health Research, Darwin, Australia (reference number 2016-2544) with recognition from the Australian Departments of Defence and Veterans Affairs Human Research Ethics Committee (142-19). This trial was registered with ClinicalTrials.gov: NCT03056391.

Patients were randomized in a 1:1 ratio to receive either regularly dosed paracetamol or no paracetamol, using computer-generated, site-specific block randomization, and treatment allocation administered through REDCap electronic software. Patients were given paracetamol (Good Manufacturing Practice (GMP)-produced Paracil, SM Pharmaceuticals, Malaysia) 1 g orally every 6 hours for 72 hours under direct observation by research personnel. In the control group, paracetamol was administered when the temperature persisted above 39.5°C for more than 30 minutes despite

tepid sponging, or if the treating clinicians deemed it necessary. All patients were treated with artesunate and/or oral artemether-lumefantrine for malaria in adherence to local guidelines.

AKI was classified according to the Kidney Disease: Improving Global Outcomes (KDIGO) criteria, excluding the urine output criteria, as this information was not routinely collected. On enrollment, venous blood samples were collected for standard hematology and biochemistry analysis. Alanine aminotransferase (ALT) measurements were monitored on admission, day 3, day 7, and day 28 for signs of hepatotoxicity. Additional ALT measurements may have been performed at the discretion of the treating clinician. Peripheral blood parasitemia was evaluated by research microscopists at enrollment and every 6 hours until two consecutive negative smears were obtained. Serum creatinine levels were assessed at enrollment, then every 12 hours for up to 72 hours, followed by measurements at days 7 and 28. The creatinine analysis was conducted in real-time at the accredited enrolling hospitals using an automated modified Jaffe alkaline picrate method (Architect c8000 Chemistry Analyzer, Abbot), with the assay traceable to the Isotope Dilution Mass Spectrometry (IDMS) standard.

For patients enrolled between 8 am and 5 pm, frequent plasma paracetamol samples were collected, where plasma samples were collected upon admission, before the first dose, and at 0.5, 1.5, 2.5, and 4 hours following the first dose of paracetamol. Subsequent samples were collected every 6 hours before each dose for 72 hours, and then at 72.5, 73.5, 74.5, 76, 78, and 84 hours after the initial administration of paracetamol. For patients enrolled outside of these working hours and those in the control group, paracetamol samples were collected using a sparse sampling schedule, with samples collected at enrollment and subsequently every 6 hours for 72 hours. All plasma samples were stored at Queen Elizabeth Hospital laboratory (-80°C), then shipped on dry-ice to Menzies School of Health Research and stored at -80°C. The samples were then transferred to the Australian Defence Force Malaria and Infectious Disease Institute (ADFMIDI, Brisbane, Australia) on dry-ice and stored at -80°C until drug analysis. Plasma paracetamol was quantified using a liquid chromatography-tandem mass spectrometry (LC-MS/MS) method at ADFMIDI. The LC-MS/MS method was based on previously published method. The lower limit of quantification (LLOQ) was 0.050 μg/mL, using 50 μL of plasma, with an inter-assay coefficient of variation of 4.4% (n=60) and an inaccuracy of 0.5%.

S2. Prior model selection and implementation

The primary reason for implementing the prior approach was that the predominantly trough sampling design resulted in the standard modelling approach being unable to reliably characterize the structural pharmacokinetic (PK) model, leading to model misspecification. Three candidate prior models were identified from the literature based on their structural similarity to the expected PK behavior of paracetamol in the current study population. Model 1 was developed in patients with *P. falciparum* malaria, representing the most clinically similar population, while Models 2 and 3 were developed in healthy volunteers. All three candidate models were evaluated for their suitability using the following assessments:

1. Objective Function Value (OFV) comparison using MAXEVAL=0

All three candidate prior models were evaluated by applying each prior model to the current dataset with all parameters fixed to their reported estimates without re-estimation (MAXEVAL=0) and computing the OFV. This served as a measure of predictive performance for each candidate prior model. Model 1 demonstrated the lowest OFV (73,359), with substantially higher OFVs for Model 2 and Model 3 (ΔOFV = +5,895 and +4,082, respectively), providing strong objective evidence for Model 1 superiority.

Model	Study population	OFV	ΔOFV
Model 1 - Wattanakul et al. 2016	Malaria patients	73,359	Reference
Model 2 - Langeskov et al. 2022	Healthy volunteers	79,254	+5,895
Model 3 - Jiang et al. 2019	Healthy volunteers	77,441	+4,082

2. η-distribution plots

For each parameter with inter-individual variability (IIV), the observed eta density was compared against the reference normal distribution derived from the prior OMEGA estimates.

Model 1 - Wattanakul et al. 2016

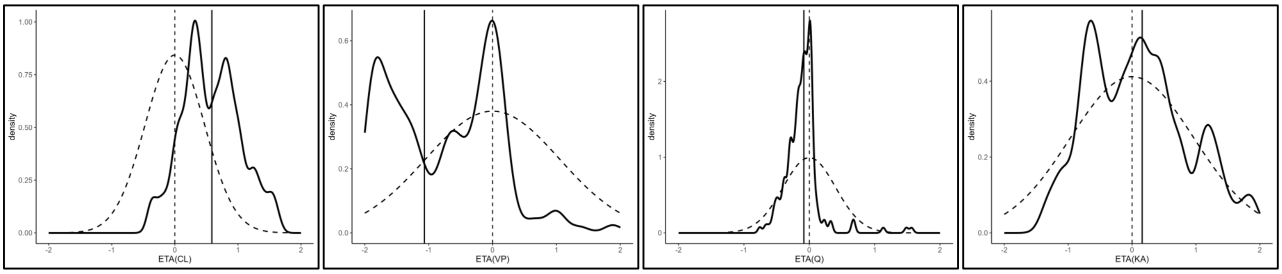

Model 2 - Langeskov et al. 2022

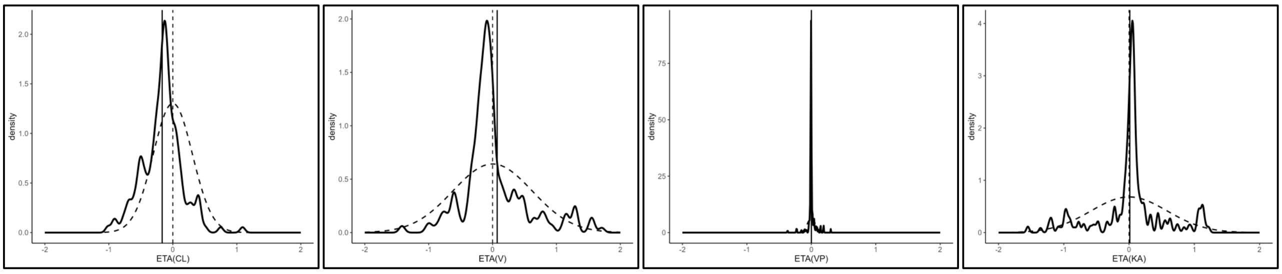

Model 3 - Jiang et al. 2019

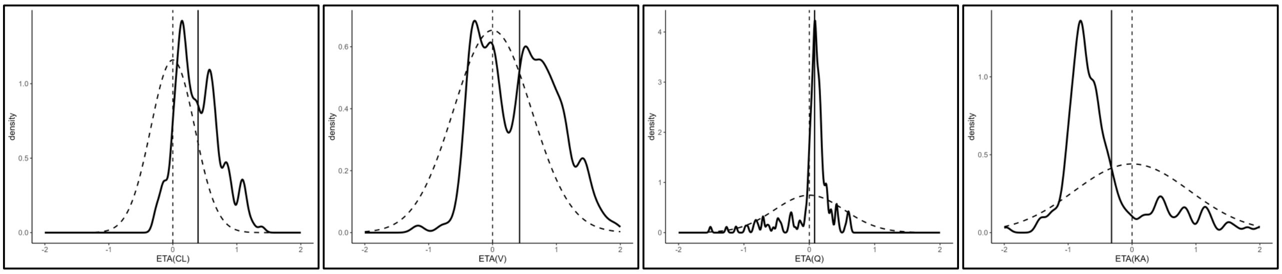

Model 2 showed high shrinkage across all parameters, with ETA(VP) density peaks exceeding 75, suggesting the prior variability estimates were poorly suited to the current population and data, preventing meaningful differentiation between individuals. For ETA(CL), Models 1 and 3 showed similar patterns with a slight rightward shift, indicating mild underestimation of clearance by the prior, with Model 3 showing slightly greater shrinkage. For ETA(V), Model 3 showed a broad distribution with low shrinkage, indicating the data adequately informed individual volume estimates. For ETA(VP), Model 1 showed a wide, leftward-shifted distribution with low shrinkage, indicating prior overestimation of peripheral volume, though individual estimates remained data-informed. For ETA(Q), both Models 1 and 3 showed high shrinkage, consistent with trough-dominated sampling being unable to inform intercompartmental clearance. For ETA(KA), Model 1 showed a reasonable distribution with low shrinkage, while Model 3 showed a leftward-shifted distribution with higher shrinkage, suggesting prior overestimation of absorption rate.

3. External visual predictive checks (VPCs)

External VPCs were generated by simulating from each prior model with parameters fixed at reported estimates (MAXEVAL=0) without re-fitting to the new dataset. Simulated concentration percentiles (5th, 50th, and 95th) were compared against observed data from the current study population. Model 1 (left) showed reasonable alignment between observed and simulated percentiles compared to the other models, with prediction intervals that reflected the variability in

the study population. Models 2 (center) and 3 (right) produced substantially narrower prediction intervals, with the observed percentiles frequently falling outside the corresponding simulated percentile bounds, indicating that both models considerably underestimated the variability in the study population and did not adequately characterize the observed concentration-time profiles.

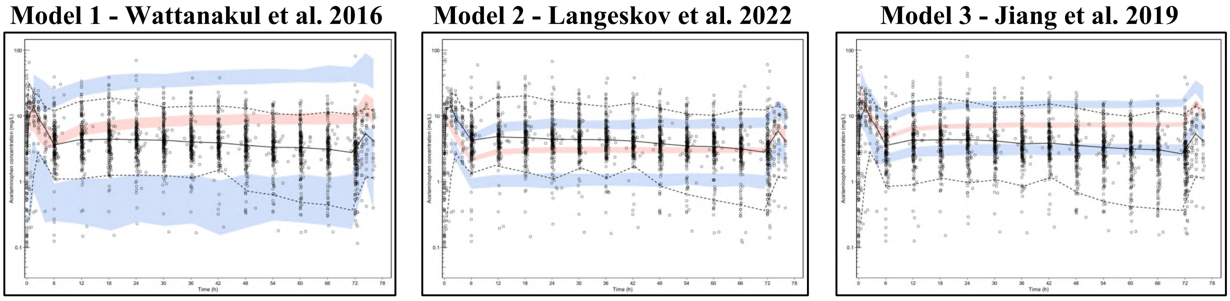

External VPCs for Model 1 (left), Model 2 (center), and Model 3 (right). Open circles represent observed paracetamol concentrations. Solid and dashed black lines represent the 50th, 5th, and 95th percentiles of observations. Shaded areas represent the 95% confidence intervals of simulated percentiles.

The results from OFV comparison, eta distribution evaluation, and external VPC consistently supported Model 1 as the most appropriate reference PK model for the current study population. Although Model 1 showed a slight rightward shift in ETA(CL) and a leftward shift in ETA(VP), these modest differences in clearance and peripheral volume between the prior and current study population are likely attributable to differences in body size or disease severity between the two populations.

PRIOR implementation approach

Following selection of Model 1 as the reference prior, three prior implementation approaches were evaluated: (1) informative prior on both θ and ω, (2) informative prior on θ with non-informative prior on ω, and (3) informative prior on θ with ω estimated freely from the current data. The third approach was motivated by the fact that the current dataset was substantially larger than the prior model in terms of number of patients, samples, and observed variability, suggesting that this population likely has different variability characteristics that are better captured by independently estimating interindividual variability from the current data rather than using variability estimates from the prior population. This approach provided the best model fit in terms of both OFV (OFV = 1,336, 1,135, and 1,068 for approaches 1, 2, and 3, respectively) and visual predictive check performance, and was therefore selected as the model approach for subsequent covariate investigation.

PRIOR approach	OFV	Selected
(1) Informative PRIOR on both THETA and OMEGA	1,336	No
(2) Informative PRIOR on THETA with non-informative PRIOR on OMEGA	1,135	No
(3) Informative PRIOR on THETA with OMEGA estimated freely	1,068	Yes

S3. Time-to-event modeling of fever clearance

Fever clearance time was characterized using a parametric time-to-event modeling framework. Two fever clearance endpoints were analyzed independently: FCT-A, defined as the time from enrollment to the first recorded body temperature below 37.5°C, and FCT-B, defined as the time from enrollment to body temperature falling below 37.5°C and remaining below this threshold for at least 24 hours. Time-to-event data from patients in both the paracetamol and control arms were modeled simultaneously using NONMEM with the Laplacian estimation method. The event was described using a hazard function. Four parametric hazard functions were evaluated i.e., exponential, Gompertz, Weibull, and log-logistic as defined below:

$$h(t) = \lambda \quad \text{(Exponential)}$$

$$h(t) = \lambda \cdot e^{\gamma \cdot t} \quad \text{(Gompertz)}$$

$$h(t) = \lambda \cdot \gamma \cdot (\lambda \cdot t)^{\gamma - 1} \quad \text{(Weibull)}$$

$$h(t) = \frac{\lambda \cdot \gamma \cdot (\lambda \cdot t)^{\gamma - 1}}{1 + (\lambda \cdot t)^{\gamma}} \quad \text{(Log-logistic)}$$

Where h(t) denotes hazard at time t, which is the instantaneous rate of fever clearance at time t, λ denotes scale parameter, γ denotes shape parameter, and t denotes time in hours.

The survival function was calculated using cumulative hazard. The survival calculated here represents the probability of not having fever clearance beyond time t.

$$S(t) = e^{-\int_0^t \frac{h(t)}{dt}} \quad \text{(Survival)}$$

Where $S(t)$ denotes survival at time t and h(t) denotes hazard function.

Table S1. Parameter estimates from the time-to-event models for fever clearance time

Parameters	Population estimates [a] (%RSE) [b]	95%CI [b]
**Fever clearance time A**		
Log-logistic hazard function		
λ	0.0940 (6.9)	0.0822-0.107
γ	2.77 (4.1)	2.57-3.02
θ_{AUC72H}	0.158 (45.7)	0.0389-0.285
**Fever clearance time B**		
Log-logistic hazard function		
λ	0.0776 (7.7)	0.0649-0.0875
γ	2.37 (3.9)	2.22-2.58
θ_{AUC72H}	0.126 (50.0)	0.0243-0.247

[a] Population mean values were estimated by NONMEM. [b] Relative standard error (%RSE) and 95 % confidence interval (95%CI) were assessed by 1,000 bootstrap runs. Abbreviations: λ, scale parameter of log-logistic hazard function; γ, shape parameter of log-logistic hazard function; θ_{AUC72H}, effect of paracetamol $AUC_{0\text{-}72H}$ (per 100 mg·h/L) on the log-logistic hazard function.

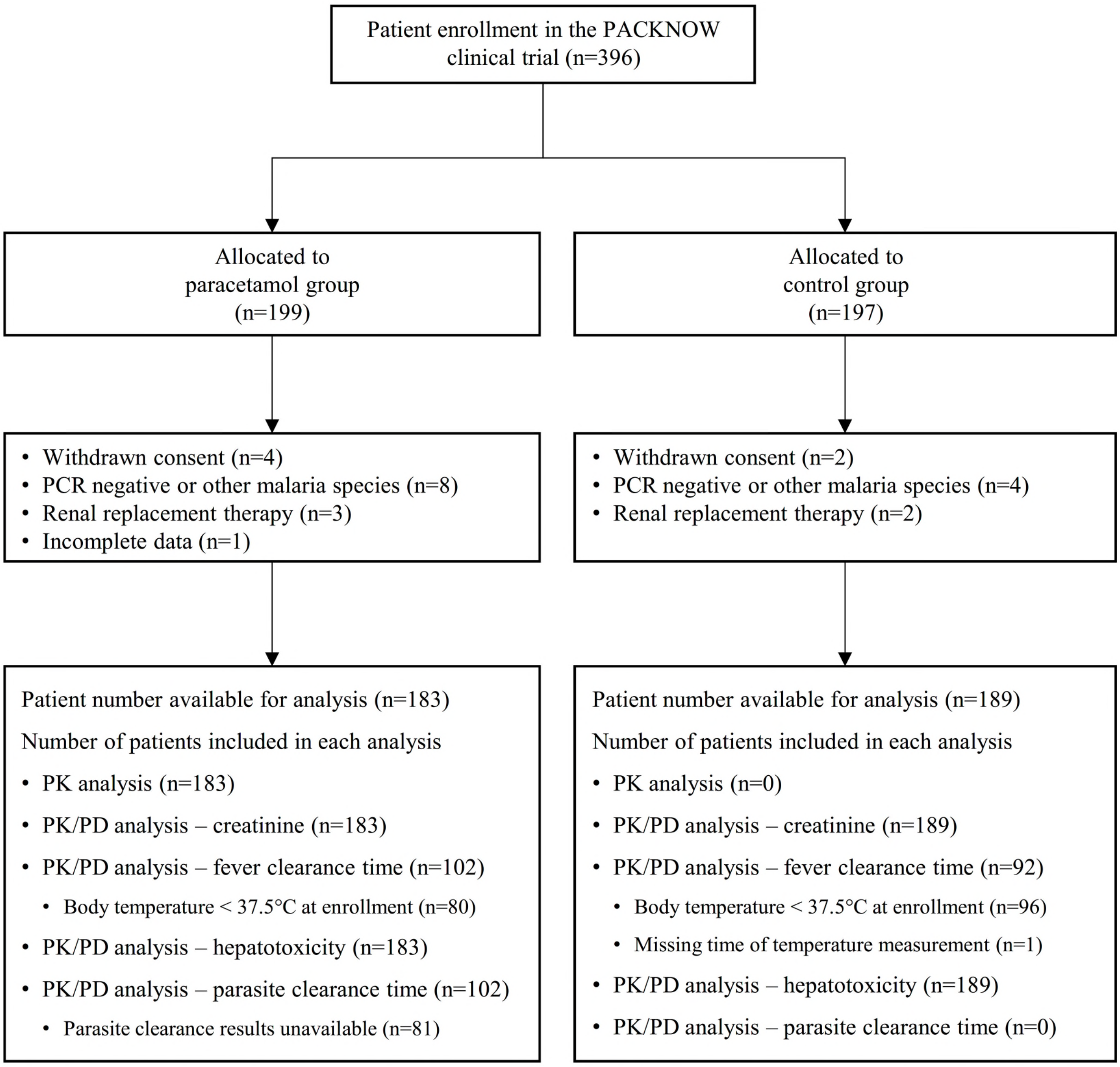

Figure S1. Participant flow diagram. Abbreviations: PACKNOW, Clinical trial entitled - Effect of Regularly Dosed Paracetamol vs No Paracetamol on Renal Function in *Plasmodium knowlesi* Malaria; PCR, polymerase chain reaction, PK analysis, pharmacokinetic analysis; PK/PD analysis, pharmacokinetic/pharmacodynamic analysis.

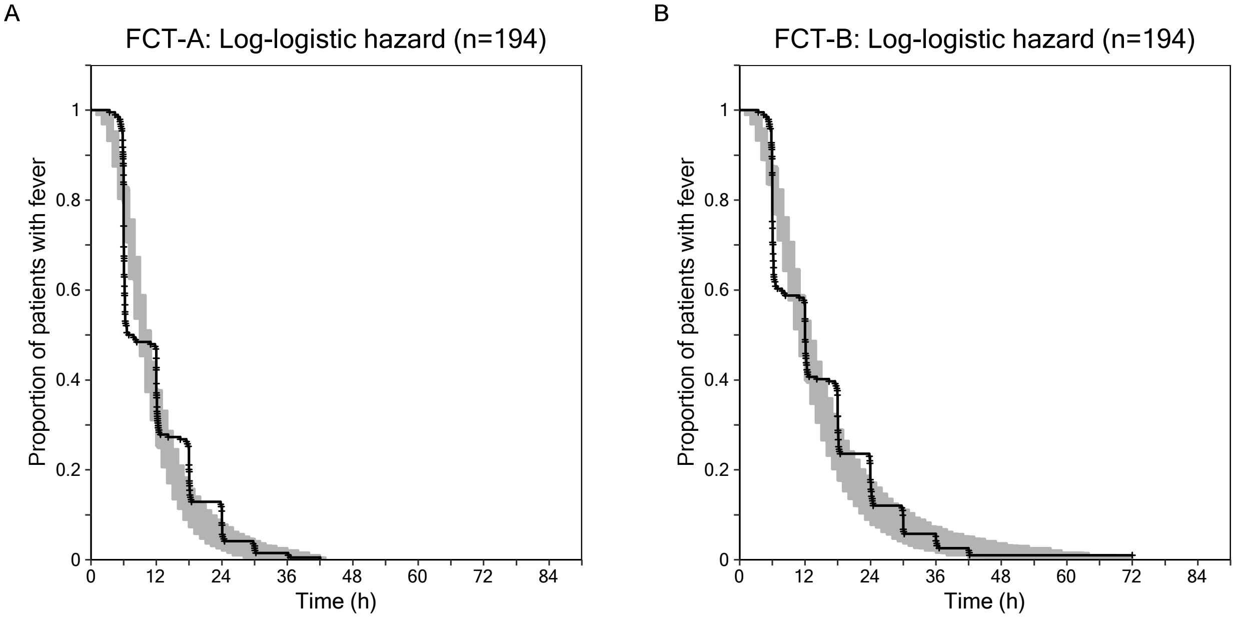

Figure S2. Visual predictive checks (n=1000) of the final time-to-event model describing paracetamol effect on fever clearance time. Visual predictive checks (n=1000) of the final time-to-event model describing FCT-A (A) and FCT-B (B). Abbreviations: FCT-A, time taken for the temperature to fall below 37.5°C; FCT-B, time taken for the temperature to fall below 37.5°C and remain there for at least 24 hours.

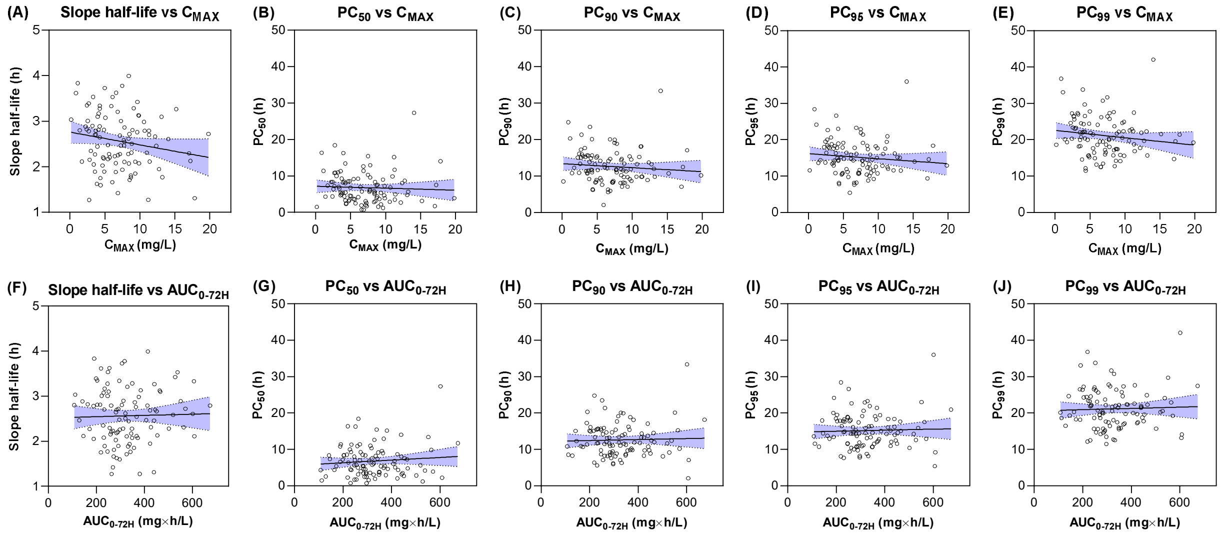

Figure S3. Relationship between paracetamol exposure and parasite clearance parameters. Slope half-life vs C_{MAX} (A), PC_{50} vs C_{MAX} (B), PC_{90} vs C_{MAX} (C), PC_{95} vs C_{MAX} (D), PC_{99} vs C_{MAX} (E), Slope half-life vs AUC_{0-72H} (F), PC_{50} vs AUC_{0-72H} (G), PC_{50} vs AUC_{0-72H} (H), PC_{50} vs AUC_{0-72H} (I), and PC_{50} vs AUC_{0-72H} (J). None of the slopes of the linear regression models were significantly different from zero (p-value > 0.05). The open circles represent the observations, black solid line represent the slope of the linear regression, and the shaded are represent the 95% confidence interval of the slope.

NONMEM code

Pharmacokinetics model

```
$PROBLEM PARACETAMOL PK – PRIOR WATTANAKUL ET AL. 2016

$INPUT ID TIME TAD AMT DV MDV EVID CMT BQL INIT_DV ARM DENSE OCC OCCN OCCN2 SEX RRT AGE WT CREAT HB HCT
PLAT TBIL DBIL ALP ALT AST LTBIL LALP LALT LGAST PT APTT INR CFHB TEMP BPARA SEVERE PCOUNT LBP MGPKG SITE
CFHBCUT LCFHB SITENEW

$DATA PACKNOW_PK_data.csv IGNORE=@

$ABBREVIATED COMRES=2

$SUBROUTINE ADVAN6 TOL=5

$PRIOR NWPRI

$MODEL
COMP = (1)                                  ; DOSE COMPARTMENT
COMP = (2)                                  ; CENTRAL COMPARTMENT
COMP = (3)                                  ; PERIPHERAL COMPARTMENT
COMP = (4)                                  ; PARACETAMOL AUC

$PK
; KAAGE-DEFINITION START                    ; DEFINE AGE EFFECT ON KA
KAAGE = (1 + THETA (9) *(AGE - 35))
; KAAGE-DEFINITION END

; KA-RELATION START
KACOV=KAAGE
; KA-RELATION END

; BETWEEN OCCASION VARIABILITY
OCC1=0
OCC2=0
OCC3=0
OCC4=0

IF (OCCN.EQ.1)   OCC1 =1                    ; OCCASION 1
IF (OCCN.EQ.2)   OCC2 =1                    ; OCCASION 2
IF (OCCN.EQ.3)   OCC3 =1                    ; OCCASION 3
IF (OCCN.EQ.4)   OCC4 =1                    ; OCCASION 4

IOVF1 = OCC1*ETA (7) + OCC2*ETA (8) + OCC3*ETA (9) +        ; INTEROCCASION VARIABILITY ON F
OCC4*ETA (10)

; EXPONENTIAL DECLINE OF F
HLF1 = THETA (7)                            ; HALF-LIFE OF THE DECLINE
KF1 = LOG (2)/HLF1                          ; RATE OF DECLINE
MINF1 = THETA (8)                           ; MINIMUM F
```

```
TEFFF1 = EXP(-KF1*TIME) *(1-MINF1) +MINF1          ; EXPONENTIAL DECLINE FUNCTION

; PK PARAMETERS
TVCL = THETA (1) *(WT/58) **0.75                   ; TYPICAL VALUE OF ELIMINATION CLEARANCE
CL = TVCL *EXP (ETA (1))                           ; INDIVIDUAL ELIMINATION CLEARANCE

TVV = THETA (2) *(WT/58)                           ; TYPICAL VALUE OF CENTRAL VOLUME OF DISTRIBUTION
V = TVV *EXP (ETA (2))                             ; INDIVIDUAL CENTRAL VOLUME OF DISTRIBUTION

TVQ = THETA (3) *(WT/58) **0.75                    ; TYPICAL VALUE OF INTERCOMPARTMENTAL CLEARANCE
Q = TVQ *EXP (ETA (3))                             ; INDIVIDUAL INTERCOMPARTMENTAL CLEARANCE

TVVP = THETA (4) *(WT/58)                          ; TYPICAL VALUE OF PERIPHERAL VOLUME OF DISTRIBUTION
VP = TVVP *EXP (ETA (4))                           ; INDIVIDUAL PERIPHERAL VOLUME OF DISTRIBUTION

TVKA = THETA (5)                                   ; TYPICAL VALUE OF ABSORPTION RATE CONSTANT
TVKA = KACOV*TVKA                                  ; EFFECT OF AGE ON ABSORPTION RATE CONSTANT
KA    = TVKA *EXP (ETA (5))                        ; INDIVIDUAL ABSORPTION RATE CONSTANT

TVF1 = THETA (6)                                   ; TYPICAL VALUE OF RELATIVE BIOAVAILABILITY
F1   = TVF1*EXP (ETA (6) +IOVF1) *TEFFF1           ; INDIVIDUAL RELATIVE BIOAVAILABILITY

; FIRST ORDER RATE CONSTANTS
K12 = KA                                           ; RATE CONSTANT FROM COMP-1 TO COMP-2
K23 = Q/V                                          ; RATE CONSTANT FROM COMP-2 TO COMP-3
K32 = Q/VP                                         ; RATE CONSTANT FROM COMP-3 TO COMP-2
K20 = CL/V                                         ; RATE CONSTANT FROM COMP-2 TO ELIMINATION
S2 = V                                             ; SCALING FACTOR (DOSE = MCG, CONC= MCG/L)
A_0(2) = INIT_DV*S2                                ; INITIALIZE COMPARTMENT WITH PRE-DOSE CONCENTRATION

; HALF-LIFE CALCULATION FOR 2-CMP
SUM = K20+K23+K32
ROOT = SQRT(SUM*SUM-4*K32*K20)
ALPHA = 0.5*(SUM+ROOT)
BETA = 0.5*(SUM-ROOT)
HTA = 0.693/(ALPHA)                                ; HALF-TIME ALPHA
HTB = 0.693/(BETA)                                 ; HALF-TIME BETA

; SECONDARY PARAMETERS
IF (NEWIND.LE.1) THEN                              ; FOR THE NEW SUBJECT
COM (1) =-1                                        ; HOLDER OF CMAX_PLASMA
COM (2) =-1                                        ; HOLDER OF TMAX_PLASMA
ENDIF

IF(AMT.GT.0) DTIM = TIME


$DES
DADT (1) = - A (1) *K12                            ; GUT COMPARTMENT
                                                   ; CENTRAL COMPARTMENT
```

```
DADT (2) = A (1) *K12 – A (2) *K20 – A (2) *K23 + A (3)
*K32                                                    ; PERIPHERAL COMPARTMENT
DADT (3) = A (2) *K23 – A (3) *K32
DADT (4) = A (2)                                        ; AUC PLASMA CONCENTRATION

AUC     = A (4)/S2                                      ; PARACETAMOL AUC
TAD2   = T-DTIM                                         ; CALCULATE TIME AFTER DOSE
CT1    = A (2)/S2                                       ; PARACETAMOL CONCENTRATION

IF (CT1.GT.COM (1)) THEN                                ; COLLECT CMAX
COM (1) =CT1                                            ; COLLECT TMAX
COM (2) =TAD2
ENDIF

$ERROR
CP = A (2)/S2
 IF (CMT.EQ.2) THEN
  IPRED = A (2)/S2                                      ; PREDICTED PARACETAMOL CONCENTRATION
  W = SQRT (SIGMA (1,1))
 ENDIF

 IF(IPRED.GT.0) THEN
  IPRED = LOG(IPRED)
 ELSE
  IPRED = 0
 ENDIF

 IF (CMT.EQ.2) THEN
  Y = IPRED + EPS (1)                                   ; ADDITIVE ERROR ON LOG SCALE
 ENDIF

 IRES = DV-IPRED
 IWRES = IRES/W

 IF(AMT.GT.0) COM (1) = 0
 IF(AMT.GT.0) COM (2) = 0

 CMAX = COM (1)
 TMAX = COM (2)

$THETA                                                  ; INITAIL ESTIMATES FOR CURRENT MODEL
(0, 9.8)                                                ; 1. TVCL
(0, 53.7)                                               ; 2. TVV
(0, 10.7)                                               ; 3. TVQ
(0, 33.2)                                               ; 4. TVVP
(0, 0.917)                                              ; 5. TVKA
(1) FIX                                                 ; 6. TVF1
(0, 10.2)                                               ; 7. HLF1
(0, 0.22)                                               ; 8. MINF1
(-0.02, -0.0187,0.043)                                  ; 9. KAAGE1
```

```
$THETAP                                  ; INITAIL ESTIMATES FROM PRIOR MODEL
10.7 FIX                                 ; 1. TVCL
45.5 FIX                                 ; 2. TVV
10.3 FIX                                 ; 3. TVQ
11.3 FIX                                 ; 4. TVVP
4.15 FIX                                 ; 5. TVKA

$OMEGA
 0 FIX                                   ; 1.IIV_CL
 0.338                                   ; 2.IIV_V
 0 FIX                                   ; 3.IIV_Q
 2.77                                    ; 4.IIV_VP
 2.13                                    ; 5.IIV_KA
 0.0662                                  ; 6.IIV_F1

$OMEGA BLOCK (1) 0.251                   ; 7.IOV_F1_OCC1
$OMEGA BLOCK (1) SAME
$OMEGA BLOCK (1) SAME
$OMEGA BLOCK (1) SAME

$THETAPV BLOCK (5) FIX                   ; VARIANCE OF THETA PRIORS
3.27E+00
0 1.50E+01
0 0 1.44E+01
0 0 0 2.33E+01
0 0 0 0 3.41E+00

$SIGMA 0.29                              ; ADDITIVE ERROR ON LOG SCALE

$ESTIMATION MAXEVAL=9999 PRINT=1 METHOD=1 INTER SADDLE_RESET=1 MCETA=500 RANMETHOD=P
```

Pharmacokinetics/Pharmacodynamics model: Effect of paracetamol on creatinine

```
$PROBLEM PK/PD: PARACETAMOL- CREATININE

$INPUT ID OCCN PTIME TIME TAD AMT DV3=DV MDV EVID CMT INITDV ARM OCC SEX RRT AGE WT CREAT HB HCT
PLAT TBIL DBIL ALP ALT AST CFHB TEMP BPARA SEVERE PCOUNT IF1 ICL IV IQ IVP IKA ITEFF1 IHLF1 IMINF AUC72
AUC72MG CONC CFHBCUT KDIGO STAGE AKI LAUC72 LAUC72MG BASECR

$DATA PKPD_creatinine_data.csv IGNORE=@

$SUBROUTINE ADVAN5 TRANS1

$MODEL
COMP = (1)                                        ; DOSE COMPARTMENT
COMP = (2)                                        ; CENTRAL COMPARTMENT
COMP = (3)                                        ; PERIPHERAL COMPARTMENT
COMP = (4)                                        ; PD: PERCENTAGE CHNAGE OF CREATININE FROM
                                                  BASELINE
$PK
; KAPPALAUC72-DEFINITION START                    ; DEFINE COVARIATE ON KAPPA
KAPPALAUC72 = (1 + THETA (4) *(LAUC72MG))
; KAPPALAUC72-DEFINITION END

; KAPPA-RELATION START
KAPPACOV=KAPPALAUC72
; KAPPA-RELATION END

; PLATEAUSTAGE-DEFINITION START                   ; DEFINE COVARIATE ON PLATEAU
IF (STAGE.EQ.0.0000E+00) PLATEAUSTAGE = 1
IF (STAGE.EQ.1.0000E+00) PLATEAUSTAGE = (1 + THETA
(4))
IF (STAGE.EQ.2.0000E+00) PLATEAUSTAGE = (1 + THETA
(5))
IF (STAGE.EQ.3.0000E+00) PLATEAUSTAGE = (1 + THETA
(6))
; PLATEAUSTAGE-DEFINITION END

; PLATEAU-RELATION START
PLATEAUCOV=PLATEAUSTAGE                           ; INDIVIDUAL F1 FROM PK MODEL
; PLATEAU-RELATION END                            ; INDIVIDUAL CL FROM PK MODEL
                                                  ; INDIVIDUAL V FROM PK MODEL
F1 = IF1                                          ; INDIVIDUAL Q FROM PK MODEL
CL = ICL                                          ; INDIVIDUAL VP FROM PK MODEL
V = IV                                            ; INDIVIDUAL KA FROM PK MODEL
Q = IQ
VP = IVP
KA = IKA                                          ; RATE CONSTANT FROM COMP-1 TO COMP-2
                                                  ; RATE CONSTANT FROM COMP-2 TO COMP-3
; FIRST ORDER RATE CONSTANTS                      ; RATE CONSTANT FROM COMP-3 TO COMP-2
K12   = KA                                        ; RATE CONSTANT FROM COMP-2 TO ELIMINATION
```

```
K23   = Q/V                                  ; SCALING FACTOR (DOSE = MCG, CONC= MCG/L)
K32   = Q/VP                                 ; INITIALIZE COMPARTMENT WITH PRE-DOSE
K20   = CL/V                                 CONCENTRATION
S2    = V
A_0(2) = INITDV*S2                           ; TYPICAL VALUE BASELINE CREATININE CHANGE
                                             ; INDIVIDUAL BASELINE CREATININE CHANGE
; PD PARAMETERS
TVBASE = THETA (1)                           ; TYPICAL VALUE KAPPA (RATE OF REDUCTION)
BASE = TVBASE + ETA (1)                      ; COVARIATE ON KAPPA
                                             ; INDIVIDUAL KAPPA (RATE OF REDUCTION)
TVKAPPA = THETA (2)
TVKAPPA = KAPPACOV*TVKAPPA                   ; TYPICAL VALUE OF PLATEAU
KAPPA = TVKAPPA + ETA (2)                    ; COVARIATE ON PLATEAU
                                             ; INDIVIDUAL PLATEAU
TVPLATEAU = THETA (3)
TVPLATEAU = PLATEAUCOV*TVPLATEAU
PLATEAU = TVPLATEAU + ETA (3)

$ERROR
CP = (A(2)/S2)/1000                          ; PREDICTED PERCENTAGE CHANGE OF CREATININE

IF (CMT.EQ.4) THEN
IPRED = (BASE-PLATEAU) *EXP(-KAPPA*TIME) +
PLATEAU
W = SQRT (SIGMA (1,1))                       ; ADDITTIVE ERROR
ENDIF

IF (CMT.EQ.4) THEN
Y = IPRED + EPS (1)
ENDIF

IRES = DV-IPRED                              ; 1. TVBASE
IWRES = IRES/W                               ; 2. TVKAPPA
                                             ; 3. TVPLATEAU
$THETA                                       ; 4. KAPPALAUC721
(0) FIX
(0, 0.0462)
(-15)                                        ; 1. IIV_BASE
(-0.166, 0.244,100000)                       ; 2. IIV_KAPPA
                                             ; 3. IIV_PLATEAU
$OMEGA
0 FIX                                        ; ADDITIVE ERROR
0.00121
254

$SIGMA 63

$ESTIMATION MAXEVAL=9999 PRINT=1 METHOD=1 INTER SADDLE_RESET=1 MCETA=100 RANMETHOD=P
```

Pharmacokinetics/Pharmacodynamics model: Effect of paracetamol on fever

```
$PROBLEM PK/PD: PARACETAMOL- FEVER CLEARANCE TIME

$INPUT ID OCCN PTIME TIME AMT DV MDV EVID CMT INIT_DV ARM SEX RRT AGE WT CREAT HB HCT PLAT TBIL DBIL
ALP ALT AST LTBIL LALP LALT LGAST PT APTT INR CFHB TEMP BPARA SEVERE PCOUNT LBP IF1 ICL IV IQ IVP IKA
AUC72 AUC72MG CONCPK FEVER0

$DATA PKPD_FCTA.csv IGNORE=@

$SUBROUTINE ADVAN=13 TOL=6

$MODEL
COMP = (1)                  ; DOSE COMPARTMENT
COMP = (2)                  ; CENTRAL COMPARTMENT
COMP = (3)                  ; PERIPHERAL COMPARTMENT
COMP = (4)                  ; FCTA HAZARD COMPARTMENT

$PK
F1 = IF1                    ; INDIVIDUAL F1 FROM PK MODEL
CL = ICL                    ; INDIVIDUAL CL FROM PK MODEL
V = IV                      ; INDIVIDUAL V FROM PK MODEL
Q = IQ                      ; INDIVIDUAL Q FROM PK MODEL
VP = IVP                    ; INDIVIDUAL VP FROM PK MODEL
KA = IKA                    ; INDIVIDUAL KA FROM PK MODEL

; FIRST ORDER RATE CONSTANTS
K12 = KA                    ; RATE CONSTANT FROM COMP-1 TO COMP-2
K23 = Q/V                   ; RATE CONSTANT FROM COMP-2 TO COMP-3
K32 = Q/VP                  ; RATE CONSTANT FROM COMP-3 TO COMP-2
K20 = CL/V                  ; RATE CONSTANT FROM COMP-2 TO ELIMINATION
S2 = V                      ; SCALING FACTOR (DOSE = MCG, CONC= MCG/L)
A_0(2) = INIT_DV*S2         ; INITIALIZE COMPARTMENT WITH PRE-DOSE
                            CONCENTRATION

; TIME TO EVENT MODEL
TVBASE = THETA (1)          ; EFFECT OF TREATMENT ARM
BASE = TVBASE*EXP (ETA (1)) ; BASELINE HAZARD
SHP = THETA (2)             ; SHAPE PARAMETER
LAM = BASE                  ; SCALE PARAMETER
BETA = THETA (3)            ; COVARIATE EFFECT
EFF    = (1+BETA*AUC72MG)   ; LINEAR EFFECT OF AUC72MG

$DES
DADT (1) = - A (1) *K12
DADT (2) = A (1) *K12 – A (2) *K23 + A (3) *K32 – A (2) *K20
DADT (3) = A (2) *K23 – A (3) *K32
DEL = 1E-6
DADT (4) = EFF*(LAM*SHP*(LAM*(T)+DEL) **(SHP-
1))/(1+(LAM*(T)+DEL) **(SHP))
```

```
$ERROR
CONC = (A(2)/S2)/1000
CHZ = A (4)                                  ; CUMULATIVE HAZARD
SUR = EXP(-CHZ)                              ; SURVIVAL FUNCTION
DELX   = 1E-6
HAZNOW = (LAM*SHP*(LAM*(TIME)+DELX) **(SHP-
1))/(1+(LAM*(TIME)+DELX) **(SHP))

IF (DV.EQ.0) Y=SUR                           ; CENSOED EVENT
IF (DV.EQ.1) Y=SUR*HAZNOW                    ; EVENT

;Sim_start                                   : ADD/REMOVE FOR SIMULATION
;  IF(ICALL.EQ.4) THEN                       ; SIMULATION
;     IF(NEWIND.NE.2) THEN                   ; NEW ID
;   CALL RANDOM(2,R)                         ; RANDOM NO FOR NEW ID
;      RA = R                                ; ASSIGN NEW RANDOM NUMBER FOR NEW ID
;    ELSE                                    ; IF SAME ID SAME RANDOM NUMBER
;      RA = RA
;    ENDIF
;
;   DV  = 0                                  ; NO EVENT OCCURS
;   TTE = 0                                  ; NO EVENT OCCURS
;
;   IF(TIME.EQ.43) TTE = 1                   ; A CENSORED EVENT
;
;   IF(RA.GT.SUR) THEN
;      DV  = 1                               ; EVENT
;      TTE = 1                               ; EVENT
;   ENDIF
;  ENDIF
;Sim_end

$THETA
(0, 0.0766,1)                                ; BASELINE HAZARD
(0, 2.37)                                    ; SHAPE
(0, 0.00126)                                 ; BETA-AUC72MG

$OMEGA
0 FIX                                        ; IIV_BASELINE HAZARD

;Sim_start                                   : ADD/REMOVE FOR SIMULATION
;$SIMULATION (5988566) (39978 UNIFORM) ONLYSIM
NOPREDICTION SUB=1000

$ESTIMATION MAXEVAL=9999 POSTHOC METHOD=1 PRINT=5 LAPLACIAN LIKE NOABORT
;Sim_end
```

Pharmacokinetics/Pharmacodynamics model: Effect of paracetamol on liver function

```
$PROBLEM PK/PD: PARACETAMOL- ALT

$INPUT ID PTIME TIME AMT BASEALT DV MDV EVID CMT BQL INITDV ARM SEX RRT AGE WT CREAT HB HCT
PLAT=DROP TBIL DBIL ALP ALT AST LTBIL LALP LALT LGAST TEMP SEVERE LBPARA CFHBCUT KDIGO STAGE AKI
IF1 ICL IV IQ IVP IKA CONC AUC72 AUC72MG BCRSR BALT

$DATA     PKPD_ALT.csv IGNORE=@

$SUBROUTINE ADVAN5 TRANS1

$MODEL
COMP = (1)                                  ; DOSE COMPARTMENT
COMP = (2)                                  ; CENTRAL COMPARTMENT
COMP = (3)                                  ; PERIPHERAL COMPARTMENT
COMP = (4)                                  ; PD COMPARTMENT [LIVER ENZYME ALT LEVEL U/L]

$PK
;;; SLOPEBALT-DEFINITION START              ; EFFECT OF BASELINE ALT ON SLOPE
IF (BALT.EQ.-99) THEN
  SLOPEBALT = 1
ELSE
  SLOPEBALT = (1 + THETA (6) *(BALT - 34))
ENDIF
;;; SLOPEBALT-DEFINITION END


;;; SLOPEAUC72MG-DEFINITION START           ; EFFECT OF PARACETAMOL AUC ON SLOPE
SLOPEAUC72MG = (1 + THETA (5) *(AUC72MG - 114.76))
;;; SLOPEAUC72MG-DEFINITION END


;;; SLOPEAGE-DEFINITION START
SLOPEAGE = (1 + THETA (4) *(AGE - 36))      ; EFFECT OF AGE ON SLOPE
;;; SLOPEAGE-DEFINITION END

;;; SLOPE-RELATION START
SLOPECOV=SLOPEAGE*SLOPEAUC72MG*SLOPEBALT
;;; SLOPE-RELATION END


;;; INTERCEPTWT-DEFINITION START
INTERCEPTWT = (1 + THETA (3) *(WT - 60))    ; EFFECT OF WEIGHT ON INTERCEPT
;;; INTERCEPTWT-DEFINITION END

;;; INTERCEPT-RELATION START
INTERCEPTCOV=INTERCEPTWT
;;; INTERCEPT-RELATION END
```

```
F1 = IF1                                 ; INDIVIDUAL F1 FROM PK MODEL
CL = ICL                                 ; INDIVIDUAL CL FROM PK MODEL
V = IV                                   ; INDIVIDUAL V FROM PK MODEL
Q = IQ                                   ; INDIVIDUAL Q FROM PK MODEL
VP = IVP                                 ; INDIVIDUAL VP FROM PK MODEL
KA = IKA                                 ; INDIVIDUAL KA FROM PK MODEL

; FIRST ORDER RATE CONSTANTS
K12 = KA                                 ; RATE CONSTANT FROM COMP-1 TO COMP-2
K23 = Q/V                                ; RATE CONSTANT FROM COMP-2 TO COMP-3
K32 = Q/VP                               ; RATE CONSTANT FROM COMP-3 TO COMP-2
K20 = CL/V                               ; RATE CONSTANT FROM COMP-2 TO ELIMINATION
S2 = V                                   ; SCALING FACTOR (DOSE = MCG, CONC= MCG/L)
                                         ; INITIALIZE COMPARTMENT WITH PRE-DOSE
A_0(2) = INITDV*S2                       CONCENTRATION

; PD-ALT
TVSLOPE = THETA (1)                      ; SLOPE OF ALT CHANGE (LN)
TVSLOPE = SLOPECOV*TVSLOPE
SLOPE = TVSLOPE + ETA (1)

TVINTERCEPT = THETA (2)                  ; INTERCEPT
TVINTERCEPT = INTERCEPTCOV*TVINTERCEPT
INTERCEPT = TVINTERCEPT + ETA (2)

$ERROR
IF (CMT.EQ.4) THEN
IPRED = INTERCEPT+SLOPE*TIME             ; PREDICTED (LN) ALT
W = SQRT (SIGMA (1,1))
ENDIF

IF (CMT.EQ.4) THEN
Y = IPRED + EPS (1)
ENDIF

IRES = DV-IPRED
IWRES = IRES/W

$THETA
(0.00249)                                ; TVSLOPE
(3.49)                                   ; TVINTERCEPT
(0.00339)                                ; INTERCEPTWT1
(-0.0125)                                ; SLOPEAGE1
(0.00326)                                ; SLOPEAUC72MG1
(-0.0108)                                ; SLOPEBALT1


$OMEGA
7.17E-06                                 ; IIV_SLOPE
```

```
0.292                                                  ; IIV_INTERCEPT

$SIGMA 0.12

$ESTIMATION MAXEVAL= 9999 PRINT=1 METHOD=1 INTER MCETA=100 RANMETHOD=P
```
